# Randomized Controlled Trials of Artificial Intelligence in Clinical Practice: Systematic Review

**DOI:** 10.2196/37188

**Published:** 2022-08-25

**Authors:** Thomas Y T Lam, Max F K Cheung, Yasmin L Munro, Kong Meng Lim, Dennis Shung, Joseph J Y Sung

**Affiliations:** 1 The Jockey Club School of Public Health and Primary Care The Chinese University of Hong Kong Hong Kong Hong Kong; 2 Stanley Ho Big Data Decision Analytics Research Centre The Chinese University of Hong Kong. Hong Kong Hong Kong; 3 Lee Kong Chian School of Medicine Nanyang Technological University Singapore Singapore; 4 Department of Medicine (Digestive Diseases) Yale School of Medicine New Haven, CT United States

**Keywords:** artificial intelligence, randomized controlled trial, systematic review, clinical, gastroenterology, clinical informatics, mobile phone

## Abstract

**Background:**

The number of artificial intelligence (AI) studies in medicine has exponentially increased recently. However, there is no clear quantification of the clinical benefits of implementing AI-assisted tools in patient care.

**Objective:**

This study aims to systematically review all published randomized controlled trials (RCTs) of AI-assisted tools to characterize their performance in clinical practice.

**Methods:**

CINAHL, Cochrane Central, Embase, MEDLINE, and PubMed were searched to identify relevant RCTs published up to July 2021 and comparing the performance of AI-assisted tools with conventional clinical management without AI assistance. We evaluated the primary end points of each study to determine their clinical relevance. This systematic review was conducted following the updated PRISMA (Preferred Reporting Items for Systematic Reviews and Meta-Analyses) 2020 guidelines.

**Results:**

Among the 11,839 articles retrieved, only 39 (0.33%) RCTs were included. These RCTs were conducted in an approximately equal distribution from North America, Europe, and Asia. AI-assisted tools were implemented in 13 different clinical specialties. Most RCTs were published in the field of gastroenterology, with 15 studies on AI-assisted endoscopy. Most RCTs studied biosignal-based AI-assisted tools, and a minority of RCTs studied AI-assisted tools drawn from clinical data. In 77% (30/39) of the RCTs, AI-assisted interventions outperformed usual clinical care, and clinically relevant outcomes improved with AI-assisted intervention in 70% (21/30) of the studies. Small sample size and single-center design limited the generalizability of these studies.

**Conclusions:**

There is growing evidence supporting the implementation of AI-assisted tools in daily clinical practice; however, the number of available RCTs is limited and heterogeneous. More RCTs of AI-assisted tools integrated into clinical practice are needed to advance the role of AI in medicine.

**Trial Registration:**

PROSPERO CRD42021286539; https://www.crd.york.ac.uk/prospero/display_record.php?RecordID=286539

## Introduction

### Background

Artificial intelligence (AI) was first described in the 1950s as a theory of human intelligence being exhibited by machines, including but not limited to learning, reasoning, and problem-solving [1]. With an exponential increase of computational power, reduced cost of data storage, improved algorithmic sophistication, and increased availability of health data from electronic health records, the era of AI has arrived in different specialties of medicine [2-4]. AI-assisted tools have been successfully applied in various clinical settings to assist diagnosis [5], improve therapy [6], and predict risk of mortality [7]. To date, 64 AI-powered medical devices and algorithms have been approved by the Food and Drug Administration in the United States [8].

The number of AI-related articles (using the Medical Subject Headings term, “artificial intelligence” as the search keyword) in the health care literature has increased dramatically from 6802 articles in 2016 to 21,160 in 2020. However, only a minority of these are prospective clinical studies, and there are few randomized controlled trials (RCTs). Several systematic reviews have been conducted to summarize the performance of recent AI-assisted tools in specific clinical settings, such as AI-assisted adenoma detection during colonoscopy [9], AI-assisted mammography in detecting breast cancer [10], AI-assisted intracranial hemorrhage recognition on computed tomography head imaging [11], AI-assisted glycemic control for patients with diabetes, and AI-assisted diagnosis of diabetes and its related complications [12]. A recent systematic review examined all studies of AI application in clinical practice, but was limited by restriction to English language and only searching full manuscripts published between January 2010 and May 2020 [13].

### Objectives

To date, no systematic review has been restricted to RCTs regarding the clinical performance of AI-assisted tools in real-life practice. As RCTs represent the best clinical evidence to examine the effects of an intervention while controlling for unmeasured confounding factors, a comprehensive search of all RCTs studying AI-assisted tools in clinical practice would provide information regarding areas of opportunity for AI to affect real-world patient care [14]. We conducted a systematic review of all RCTs studying AI-assisted tools in clinical care.

## Methods

### Search Strategy

The systematic review was conducted following the updated PRISMA (Preferred Reporting Items for Systematic Reviews and Meta-Analyses) 2020 guidelines [15]. We comprehensively searched CINAHL, Cochrane Central, Embase, MEDLINE, and PubMed from inception to July 14, 2021, to identify RCTs of AI-based tools across all medical specialties. Details of the full search strategy are provided in Multimedia Appendix 1. The search strategy included a combination of keywords and standardized Medical Subject Headings terms: “Artificial Intelligence,” “Deep Learning,” “Computer-Assisted Diagnosis,” “Computer Assisted Diagnosis,” “Computational Intelligence,” “Computer Reasoning,” “Computer Vision System,” “Knowledge Acquisition,” “Knowledge Representation,” “Machine Intelligence” or “Machine Learning” or “Transfer Learning” or “Hierarchical Learning.” The search was limited to RCTs. We also hand searched the references of the included studies to identify additional studies of interest. To include as many previous endeavors in this research area as possible, our search was not limited to peer-reviewed information. Conference abstracts and preprints were also included. The authors had no funding source for this study. This study was registered on PROSPERO (CRD42021286539).

### Study Selection

After removing duplicates, two study authors (TYTL and MFKC) independently screened the title, abstract, and full text (if available) of each article to determine their eligibility. Unresolved disagreements were resolved by consulting the senior author (JJYS). Discrepancies were resolved by consensus. The complete manuscript was downloaded if the study met the inclusion criteria. We included studies that met the following inclusion criteria: (1) application of AI-assisted tools in clinical practice, which is defined as diagnosis, treatment, and prognostication on medical conditions that are seen and managed in daily clinical practice in hospitals or clinics. This does not include cellular or tissue cultures, animal studies, or experimental conditions such as induced cardiac arrhythmia and metabolic abnormalities. We classified the tool as AI-powered if the expressions, “artificial intelligence,” “AI,” “machine learning,” “deep learning,” “deep neural network,” and “neural network” were used to describe the tool within the articles or other publicly available information resources; (2) patients or health care providers must be involved; (3) study design must be an RCT; and (4) control group must be without AI assistance. The exclusion criteria were as follows: (1) studies without implementation of clinical AI-assisted tools for patient management; (2) studies that were not conducted as original RCTs, for example, secondary analysis of a published RCT; and (3) clinical outcome not clearly defined. Reasons for exclusion were also recorded.

### Data Extraction

After identifying relevant studies, the same two authors (TYTL and MFKC) independently extracted the data from each included study. Study design (racial information, sample size, RCT setting and design, and AI intervention and control) and AI-assisted tool characteristics (AI-assisted tool name, AI subtype, data type, and training and validation data) were documented. If AI development–related data were not available in the included articles, previously published articles of the same AI-assisted tool were reviewed to obtain the relevant information. Study end points (performance metrics used in primary and secondary end points) were listed. Clinically relevant end points were defined as whether the AI-assisted tools led to subsequent clinical interventions focusing on specific end points: (1) further diagnostic workup and investigation of the medical conditions, (2) changes in treatment strategy, (3) requirement of hospitalization, (4) escalation of care to the intensive care unit, and (5) influence on survival and mortality. Two independent researchers (TYTL and MFKC) resolved disagreements through discussion. If there were unresolved disagreements, consultation from senior author (JJYS) was sought.

### Assessment of Risk of Bias

Risk of bias was assessed using the Cochrane risk-of-bias tool for randomized trials [16]. We specifically assessed the risk of bias of randomization process, deviations from intended interventions, missing outcome data, measurement of the outcome, and selection of the reported results. The overall risk of bias was classified as low, some concerns, or high.

## Results

### Overview

**Figure 1:**
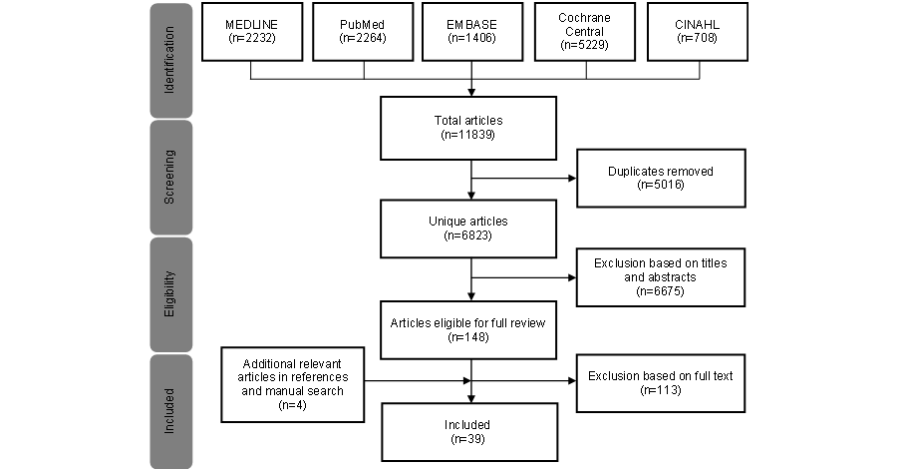
PRISMA (Preferred Reporting Items for Systematic Reviews and Meta-Analyses) flow diagram.

The search performed on July 14, 2021, yielded 11,839 articles (n=2232, 18.85% from MEDLINE; n=1406, 11.88% from Embase; n=2264, 19.12% from PubMed; n=5229, 44.17% from Cochrane Central; and n=708, 5.98% from CINAHL); of these, 6823 (57.63%) were screened after removal of duplicates (n=5016, 42.37%). After screening the titles and abstracts, 6676 articles were excluded, because they did not fulfill the inclusion criteria. A total of 147 full manuscripts were individually assessed, of which 34 (23.1%) met the inclusion criteria. In addition, 4 more articles were identified by examining the references of the listed articles and manual searches (Figure 1). A total of 39 articles were included in this systematic review [6,17-54] as listed in Table 1.

**Table 1:** Publications.

Author (publication year)	Title	Country	Article type	Specialty
El Solh et al [17], 2009	Predicting optimal CPAP by neural network reduces titration failure: a randomized study	The United States	Original article	Respiratory medicine
Shimabukuro et al [18], 2017	Effect of a machine learning-based severe sepsis prediction algorithm on patient survival and hospital length of stay: a randomised clinical trial	The United States	Original article	Anesthesiology
Labovitz et al [6], 2017	Using artificial intelligence to reduce the risk of nonadherence in patients on anticoagulation therapy	The United States	Original article	Neurology
Gracey et al [19], 2018	Improving medication adherence by better targeting interventions using artificial intelligence-a randomized control study	The United States	Abstract	Family medicine
Liu et al [20], 2018	Evaluating the impact of an integrated computer-based decision support with person-centered analytics for the management of hypertension: a randomized controlled trial	China	Abstract	Cardiology
Vennalaganti et al [21], 2018	Increased detection of Barrett’s esophagus–associated neoplasia using wide-area trans-epithelial sampling: a multicenter, prospective, randomized trial	The United States	Original article	Gastroenterology and hepatology
Biester et al [22], 2019	DREAM5: An open-label, randomized, cross-over study to evaluate the safety and efficacy of day and night closed-loop control by comparing the MD-Logic automated insulin delivery system to sensor augmented pump therapy in patients with type 1 diabetes at home	Germany, Israel, and Slovenia	Original article	Endocrinology, diabetes, and metabolism
Pouska et al [23], 2019	The use of HPI (Hypotension probability indicator) during major intracranial surgery; preliminary results of a prospective randomized trial	Czech Republic	Abstract	Anesthesiology
Lin et al [24], 2019	Diagnostic efficacy and therapeutic decision-making capacity of an artificial intelligence platform for childhood cataracts in Eye Clinics: a multicenter randomized controlled trial	China	Original article	Ophthalmology
Kamdar et al [25], 2019	A randomized controlled trial of a novel artificial intelligence–based smartphone application to optimize the management of cancer-related pain	The United States	Abstract	Clinical oncology
Persell et al [26], 2020	Effect of home blood pressure monitoring via a smartphone hypertension coaching application or tracking application on adults with uncontrolled hypertension: a randomized clinical trial	The United States	Original article	Cardiology
Voss et al [27], 2019	Effect of wearable digital intervention for improving socialization in children with autism spectrum disorder: a randomized clinical trial	The United States	Original article	Psychiatry
Wang et al [28], 2019	Real-time automatic detection system increases colonoscopic polyp and adenoma detection rates: a prospective randomised controlled study	China	Original article	Gastroenterology and hepatology
Wu et al [29], 2019	Randomised controlled trial of WISENSE, a real-time quality improving system for monitoring blind spots during esophagogastroduodenoscopy	China	Original article	Gastroenterology and hepatology
Pavel et al [30], 2020	A machine-learning algorithm for neonatal seizure recognition: a multicentre, randomised, controlled trial	Ireland, the Netherlands, Sweden, and the United Kingdom	Original article	Neurology
Alfonsi et al [31], 2020	Carbohydrate counting app using image recognition for youth with type 1 diabetes: pilot randomized control trial	Canada	Original article	Endocrinology, diabetes, and metabolism
Auloge et al [32], 2020	Augmented reality and artificial intelligence–based navigation during percutaneous vertebroplasty: a pilot randomised clinical trial	France	Original article	Orthopedics and traumatology
Avari et al [33], 2020	Safety and feasibility of the PEPPER adaptive bolus advisor and safety system; a randomized control study	The United Kingdom and Spain	Original article	Endocrinology, diabetes, and metabolism
Chen et al [34], 2020	Comparing blind spots of unsedated ultrafine, sedated, and unsedated conventional gastroscopy with and without artificial intelligence: a prospective, single-blind, 3-parallel-group, randomized, single-center trial	China	Original article	Gastroenterology and hepatology
Gong et al [35], 2020	Detection of colorectal adenomas with a real-time computer-aided system (ENDOANGEL): a randomised controlled study	China	Original article	Gastroenterology and hepatology
Liu et al [36], 2020	The single-monitor trial: an embedded CADe system increased adenoma detection during colonoscopy: a prospective randomized study	China	Original article	Gastroenterology and hepatology
Nicolae et al [37], 2020	Conventional vs machine learning-based treatment planning in prostate brachytherapy: results of a phase I randomized controlled trial	Canada	Original article	Clinical oncology
Repici et al [38], 2020	Efficacy of real-time computer-aided detection of colorectal neoplasia in a randomized trial	Italy	Original article	Gastroenterology and hepatology
Su et al [39], 2020	Impact of a real-time automatic quality control system on colorectal polyp and adenoma detection: a prospective randomized controlled study (with videos)	China	Original article	Gastroenterology and hepatology
Wang et al [40], 2020	Lower adenoma miss rate of computer-aided detection-assisted colonoscopy vs routine white-light colonoscopy in a prospective tandem study	China	Original article	Gastroenterology and hepatology
Wang et al [41], 2020	Effect of a deep-learning computer-aided detection system on adenoma detection during colonoscopy (CADe-DB trial): a double-blind randomised study	China	Original article	Gastroenterology and hepatology
Wijnberge et al [42], 2020	Effect of a machine learning-derived early warning system for intraoperative hypotension vs standard care on depth and duration of intraoperative hypotension during elective noncardiac surgery: the HYPE randomized clinical trial	The Netherlands	Original article	Anesthesiology
Weisinger et al [43], 2021	Artificial intelligence-powered non-invasive and frequency-tuned electromagnetic field therapy improves upper extremity motor function in sub-acute stroke patients: a pilot randomized controlled trial	Israel	Abstract	Neurology
Blomberg et al [44], 2021	Effect of machine learning on dispatcher recognition of out-of-hospital cardiac arrest during calls to emergency medical services: a randomized clinical trial	Denmark	Original article	Emergency medicine
Browning et al [45], 2021	The clinical effectiveness of using a predictive algorithm to guide antidepressant treatment in primary care (PReDicT): an open-label, randomised controlled trial	The United Kingdom, Spain, Germany, France, and the Netherlands	Original article	Psychiatry
Jayakumar et al [46], 2021	Comparison of an artificial intelligence-enabled patient decision aid vs educational material on decision quality, shared decision-making, patient experience, and functional outcomes in adults with knee osteoarthritis: a randomized clinical trial	The United States	Original article	Orthopedics and traumatology
Kamba et al [47], 2021	A multicentre randomized controlled trial to verify the reducibility of adenoma miss rate of colonoscopy assisted with artificial intelligence–based software	Japan	Abstract	Gastroenterology and hepatology
Luo et al [48], 2021	Artificial intelligence-assisted colonoscopy for detection of colon polyps: a prospective, randomized cohort study	China	Original article	Gastroenterology and hepatology
Rafferty et al [49], 2021	A novel mobile app (Heali) for disease treatment in participants with irritable bowel syndrome: randomized controlled pilot trial	The United States	Original article	Gastroenterology and hepatology
Repici et al [50], 2021	Artificial intelligence and colonoscopy experience: lessons from two randomised trials	Italy and Switzerland	Original article	Gastroenterology and hepatology
Strömblad et al [51], 2021	Effect of a predictive model on planned surgical duration accuracy, patient wait time, and use of presurgical resources: a randomized clinical trial	The United States	Original article	Surgery
Wu et al [52], 2021	Evaluating the effects of an artificial intelligence system on endoscopy quality and preliminarily testing its performance on detecting early gastric cancer: a randomized controlled trial	China	Original article	Gastroenterology and hepatology
Yao et al [53], 2021	Artificial intelligence-enabled electrocardiograms for identification of patients with low ejection fraction: a pragmatic, randomized clinical trial	The United States	Original article	Cardiology
Brown et al [54], 2021	Deep learning computer-aided polyp detection reduces adenoma miss rate: a US multi-center randomized tandem colonoscopy study (CADeT-CS trial)	The United States	Original article	Gastroenterology and hepatology

### Study Characteristics

**Figure 2:**
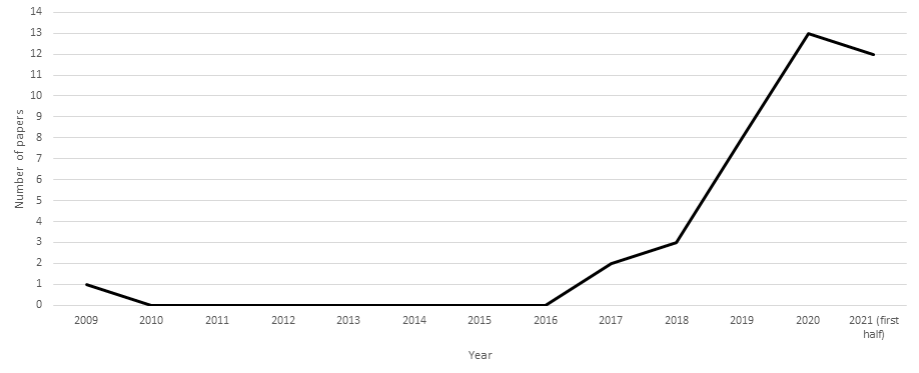
Number of randomized controlled trials of artificial intelligence–assisted medicine per year.

There were very few RCTs on AI-assisted medicine published until 2017. There was 1 RCT published in 2009, and the remaining 38 were published in the past 5 years (2 in 2017, 3 in 2018, 7 in 2019, 14 in 2020, and 12 in the first half of 2021; Figure 2).

**Figure 3:**
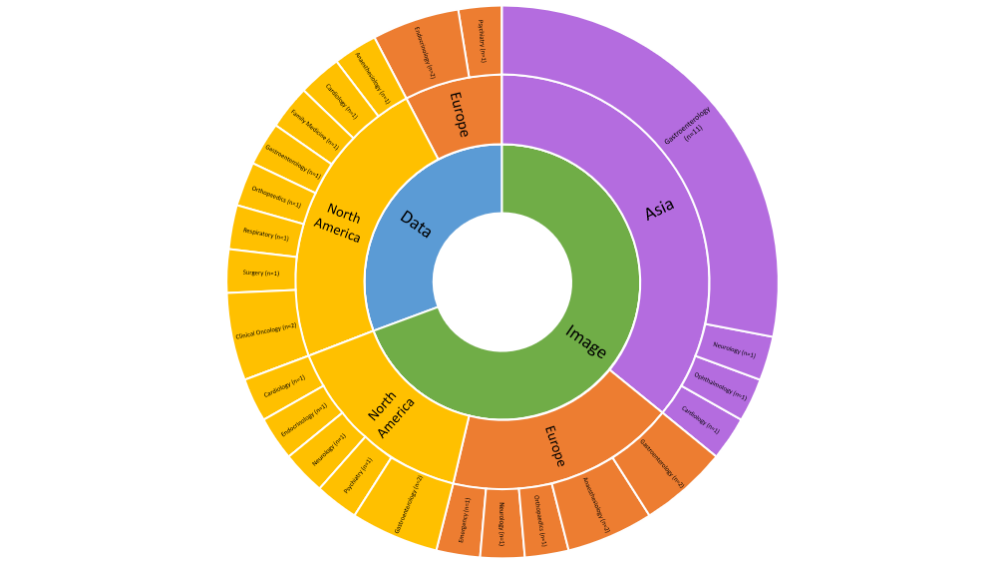
Distribution of original of publications and specialty.

These RCTs were conducted across 16 countries in North America, Europe, and Asia, with most of them conducted in the United States (13/39, 33%) and China (12/39, 31%). Furthermore, 18% (7/39) of the RCTs were published as conference abstracts only. Of these 39 publications, 16 (41%) were related to gastroenterology, whereas other specialties included anesthesiology (n=3, 7.7%), cardiology (n=3, 7.7%), endocrinology (n=3, 7.7%), psychiatry (n=2, 5%), neurology (n=3, 7.7%), orthopedics (n=2, 5%), oncology (n=2, 5%), surgery (n=1, 2.6%), ophthalmology (n=1, 2.6%), respiratory medicine (n=1, 2.6%), family medicine (n=1, 2.6%), and emergency medicine (n=1, 2.6%; Figure 3).

### Study Design

Multimedia Appendix 2 shows the study design and AI-assisted tool characteristics of each selected RCT. Most studies were single centered with a limited number of patients. Of these 39 studies, 35 (90%) had a sample size <1000 participants, 11 (28%) studies recruited fewer than 100 participants, 2 (5%) studies had a sample size of >1000 participants, and only 2 (5%) studies recruited >10,000 participants. More than half of the included RCTs (23/39, 59%) were conducted in a single center, 36% (14/39) of the studies were conducted across multiple centers, and 5% (2/39) of the studies did not mention how many centers were involved. A total of 16 open-label studies were conducted. Only 7 studies mentioned the racial information of the participants. A total of 13 blinded randomized trials were identified, of which 4 (31%) were double blinded and 9 (69%) were single blinded. The remaining 10 studies did not mention the level of blinding. Furthermore, 8 studies had a crossover study design. Most RCTs (36/39, 92%) compared the AI-assisted tools to control arms using the standard of care. Furthermore, 5% (2/39) of the studies used a sham treatment without AI assistance as the control group. A study used a mobile app without AI assistance as the control arm.

### AI-Assisted Tool Characteristics

Biosignal-based AI tools are more common than clinical data–based tools. A total of 26 AI-assisted tools were biosignal based. Endoscopic images were the most commonly used biosignal (15/26, 58%). Furthermore, 50% (13/26) of the AI-assisted tools used clinical or biochemical data for analysis (patients’ demography, self-administered questionnaire, and other relevant clinical data such as blood test results, blood pressure, and continuous positive airway pressure). No AI-assisted tool used both biosignal and clinical data combined as source data in the algorithm.

Most AI-assisted tools relied on static data (34/39, 87%) input to build the algorithm instead of dynamic data input (5/39, 13%). Static data refer to a snapshot of image or data of patients at a specific time point, whereas dynamic data are those captured continuously over a certain period during the study. For example, still images of the intestinal lumen captured during colonoscopy for AI-assisted adenoma detection are static data, whereas hourly captured vital signs and selected available laboratory tests for AI-assisted prediction of severe sepsis are dynamic data [18].

Approximately half of the studies (19/39, 49%) reported the AI-assisted tools development process. Of these, three AI-assisted tools in 8 studies, namely, GI-Genius, EndoScreener, and CC-Cruiser, were developed using data from multiple centers, whereas others were developed using data from a single center. A total of 35 studies reported the AI developer. Of these, 18 (51%) AI-assisted tools were developed by industry and 17 (49%) were developed by academic institutions.

### Study End Points

Table 2 presents the study objectives and end points. Approximately half of the studies (18/39, 46%) used diagnostic accuracy as primary end point. The most common diagnostic end point is adenoma or polyp detection rate during colonoscopy. A total of 13 studies measured treatment response after AI-assisted intervention. Quality assurance of interventions was examined in 7 studies. End point measures of 27 studies were considered clinically relevant: 19 (70%) led to further investigation, 6 (22%) indicated the need for change in treatment, 1 (4%) reported in-hospital mortality and length of hospitalization, and 1 (4%) reported hospital admission.

**Table 2:** Study objectives and end points (primary and secondary).

Author (publication year)	Study objective	Primary end point	Secondary end point	Clinical relevance
El Solh et al [17], 2009	To test the effectiveness of an ANN^a^ application for CPAP^b^ titration on the time required to achieve an optimal CPAP pressure and CPAP titration failure	Time to optimal CPAP pressure	Titration failure rate	Change of treatment
Shimabukuro et al [18], 2017	To test the use of a machine learning–based severe sepsis prediction system for reductions in average length of stay and in-hospital mortality rate	Average hospital length of stay	In-hospital mortality rate; ICU^c^ length of stay	Mortality and hospital and ICU length of stay
Labovitz et al [6], 2017	To evaluate the use of an artificial intelligence platform on mobile devices in measuring and increasing medication adherence in patients with stroke on anticoagulation therapy	Medication adherence	Nil	Nil
Gracey et al [19], 2018	To evaluate the effectiveness of using artificial intelligence to target which patients should receive interventions compared with traditional targeting approaches to improve medication adherence	Medication adherence	Nil	Nil
Liu et al [20], 2018	To assess the effects of clinical decision support system of graph-based machine learning algorithms on blood pressure management and economic burden of disease	Blood pressure reduction in patients with hypertension	Economic burden	Change of treatment
Vennalaganti et al [21], 2018	To evaluate the use of WATS^d^ as an adjunct to biopsy sampling for the detection of HGD^e^ or EAC^f^ in a referral population with BE^g^	Rate of detection of HGD or EAC	Neoplasia detection rates based on the procedure order (WATS vs biopsy sampling first) of each procedure separately and the additional time required for WATS	Further investigation
Biester et al [22], 2019	To evaluate the safety and efficacy of 60-hour glucose control using the MD-Logic system in individuals with type 1 diabetes at home for day and night use, particularly without remote monitoring	Percentage of glucose sensor readings within 70 to 180 mg/dL (3.9-10 mmol/L)	Percentage of glucose sensor readings <60 to 70 mg/dL (3.3-3.9 mmol/L), percentage of glucose sensor readings >180 to 240 mg/dL (10-13.3 mmol/L), average and SD of glucose sensor readings, and overnight percentage of readings (“overnight” defined as 11:00 PM-7:00 AM) <70 mg/dL (3.9 mmol/L)	Further investigation
Pouska et al [23], 2019	To assess the use of HPI^h^ to avoid hypotension in major intracranial surgery	Number of hypotension events; duration of hypotension events	Number of hypotension events in maintenance phase of anesthesia	Further investigation
Lin et al [24], 2019	To compare the diagnostic efficacy and treatment decision-making capacity between CC-Cruiser and ophthalmologists in real-world clinical settings	Accuracy of the diagnosis normal lens versus cataract	Evaluation of the disease severity; time required for making the diagnosis; patient satisfaction	Further investigation
Kamdar et al [25], 2019	To examine the impact of ePAL on cancer pain severity, attitudes toward cancer pain, and health care use	Pain severity	Attitudes toward cancer treatment (Barriers Questionnaire II); anxiety (General anxiety Disorder-7); pain-related hospital admissions	Hospitalization
Persell et al [26], 2020	To evaluate the effectiveness of an artificial intelligence smartphone coaching app to promote hypertension self-management	SBP^i^ measured at 6 months	Self-reported medication adherence; home monitoring and self-management practices; self-efficacy related to BP^j^ and BMI; self-reported health behaviors	Change of treatment
Voss et al [27], 2019	To test the efficacy of a wearable machine learning tool for intervention on a core ASD^k^ deficit in the natural home environment	SRS-II^l^ total score; Vineland Adaptive Behavioural Scales, Second edition; Developmental Neuropsychological Assessment, Second edition; Emotion Guessing Game	Moderator analysis; child behavior checklist; and the Vineland Adaptive Behavioural Scales, Second edition adaptive composite score	Nil
Wang et al [28], 2019	To investigate whether a high-performance real-time automatic polyp detection system can increase polyp and ADRs^m^ in the real clinical setting	ADR	PDR^n^; mean number of polyps detected per colonoscopy; mean number of adenomas detected per colonoscopy; rate of false positives and false negatives	Further investigation
Wu et al [29], 2019	To evaluate the effectiveness of WISENSE to monitor blind spots, time the procedure, and automatically generate photo documentation during EGD^o^ and thus raise the quality of everyday endoscopy	Blind spot rate	Inspection time; completeness of photo documentation generated by endoscopists; completeness of photo documentation generated by WISENSE in WISENSE group; completeness of photo documentation generated by WISENSE and endoscopists in WISENSE group; the percentage of patients being ignored in each site	Further investigation
Pavel et al [30], 2020	To evaluate the performance of the ANSeR^p^ algorithm in real time by assessing the diagnostic accuracy for the detection of neonatal electrographic seizures with and without the use of ANSeR as a support tool for clinicians at the cot side	Diagnostic accuracy (sensitivity, specificity, and false detection rate) of health care professionals to identify neonates with electrographic seizures and seizure hours with and without the support of the ANSeR algorithm	Summary measures of seizure burden (total seizure burden, maximum hourly seizure burden, and median seizure duration); number of inappropriate antiseizure medications given	Change of treatment
Alfonsi et al [31], 2020	To test the app’s usability and potential impact on carbohydrate counting accuracy	Carbohydrate counting accuracy	Quality of life for youth; self-care; patient or parent responsibility	Nil
Auloge et al [32], 2020	To evaluate technical feasibility, accuracy, safety, and patient radiation exposure granted by a novel navigational tool integrating augmented reality and artificial intelligence during percutaneous vertebroplasty of patients with vertebral compression fractures	Technical feasibility of trocar placement using augmented reality or artificial intelligence guidance	Comparison between groups A and B in terms of accuracy, procedural safety, time for trocar placement, and patient radiation exposure (dose area product and fluoroscopy time)	Nil
Avari et al [33], 2020	To evaluate the safety and efficacy of the PEPPER system compared with a standard bolus calculator	Difference in change in percentage time in range (3.9-10.0 mmol/L; 70-180 mg/dL) between the intervention arm that receives the PEPPER safety system with adaptive bolus advice and the control arm	Percentage time spent in euglycemia, hypoglycemia, and hyperglycemia; number of episodes of serious hypoglycemia; episodes of hypoglycemia within 5 hours postprandially; severe hypoglycemia (defined as a hypoglycemia event requiring third party assistance); postprandial mean area under the curve at 5 hours (expressed as mmol/L min); glycemia risk and variability measures	Change of treatment
Chen et al [34], 2020	To compare blind spots of sedated C-EGD^q^, unsedated U-TOE^r^, and unsedated C-EGD with and without the assistance of ENDOANG	The blind spot of 3 types of EGD with the assistance of ENDOANGEL	Blind spot rate of unsedated U-TOE and unsedated and sedated C-EGD with or without the assistance of ENDOANGEL; consistency between ENDOANGEL and endoscopists’ review	Further investigation
Gong et al [35], 2020	To evaluate whether the ENDOANGEL system could improve polyp yield during colonoscopy	ADR	The ADR for adenomas of different sizes (diminutive [≤5 mm], small [>5 to<10 mm], and large [≥10 mm]); locations (cecum, ascending colon, transverse colon, descending colon, sigmoid colon, and rectum); PDR; PDR for polyps of different sizes; locations; mean number of adenomas per patient; mean number of polyps per patient; withdrawal time (time spent viewing as the endoscope is withdrawn during a colonoscopy, excluding biopsy or treatment time); adverse events and serious adverse events	Further investigation
Liu et al [36], 2020	To investigate whether the integration of a CADe^s^ system into the primary monitor used during colonoscopy may increase polyp and adenoma detection without increasing physician fatigue	ADR	PDR; polyps per colonoscopy and adenomas per colonoscopy	Further investigation
Nicolae et al [37], 2020	To evaluate the noninferiority of day 30 dosimetry between a machine learning–based treatment planning system for prostate low-dose-rate brachytherapy and the conventional manual planning technique	The 1-month postoperative follow-up results between expert-planned low-dose-rate treatments (conventional) and the PIPA^t^ machine learning approach	The efficiency of the PIPA approach in a standardized preoperatively planned workflow; total treatment planning time; need and extent of modifications	Nil
Repici et al [38], 2020	To assess the safety and efficacy of a CADe system in detection of colorectal neoplasias during real-time colonoscopy	ADR	Proximal ADR; total number of polyps detected; sessile serrated lesion detection rate; mean number of adenomas per colonoscopy; cecal intubation rate; withdrawal time	Further investigation
Su et al [39], 2020	To develop an automatic quality control system and assess whether it could improve polyp and adenoma detection in clinical practice	ADR	PDR; mean number of adenomas detected per colonoscopy; mean number of polyps detected per colonoscopy; withdrawal time (biopsy time was excluded by stopping the clock); adequate bowel preparation rate, defined as the percentage of colonoscopies with each segmental BBPS^u^ score 2	Further investigation
Wang et al [40], 2020	To compare adenoma miss rates of CADe colonoscopy vs routine white-light colonoscopy.	Adenoma miss rate	Polyp miss rate; miss rate of advanced adenomas; sessile serrated adenoma or polyps; patient miss rate; ADR for the first pass; adenoma per colonoscopy; polyp per colonoscopy	Further investigation
Wang et al [41], 2020	To perform a double-blinded study using a sham control to more rigorously assess the effectiveness of a CADe system for improving detection of colon adenomas and polyps. We also aimed to analyze the characteristics of polyps missed by endoscopists	ADR	PDR; number of polyps per colonoscopy; number of adenomas per colonoscopy; sensitivity; specificity of the 3 skilled endoscopists	Further investigation
Wijnberge et al [42], 2020	To test whether the clinical application of the early warning system in combination with a hemodynamic diagnostic guidance and treatment protocol reduces intraoperative hypotension	Time-weighted average of hypotension during surgery	Incidence of hypotension (the number of hypotensive events per patient): total time with hypotension and percentage of time spent with hypotension during surgery; incidence of hypertension (the number of hypotensive events per patient): total time with hypertension and percentage of time spent with hypertension during surgery	Further investigation
Weisinger et al [43], 2021	To explore the benefit of BrainQ’s novel and noninvasive, artificial intelligence–powered, frequency-tuned ELF-EMF^v^ treatment (BQ) in improving upper extremity motor function in a population with subacute ischemic stroke	Fugl-Meyer Assessment-Upper Extremity score	Modified Rankin Scale; Action Research Arm Test; Box and Block Test; NIHSS^w^	Nil
Blomberg et al [44], 2021	To examine how a machine learning model trained to identify OHCA^x^ and alert dispatchers during emergency calls affected OHCA recognition and response	Rate of dispatchers’ recognition of subsequently confirmed OHCA	Dispatchers’ time to recognition of OHCA; rate of DA-CPR^y^	Change of treatment
Browning et al [45], 2021	To assess the clinical effectiveness of using a predictive algorithm based on behavioral tests of affective cognition and subjective symptoms and to guide antidepressant treatment	Treatment response of depression symptoms	Change in anxiety scores at week 8 (measured using the Generalized Anxiety Disorder Assessment, 7 item version [30]); remission of depression at week 8 (defined as QIDS-SR-16^z^ score of ≤5); change in the individual item scores from the QIDS-SR-16 measuring restlessness and sadness at week 8; change in symptoms of depression (treated as a continuous variable) across 12 months (measured using QIDS-SR-16); change in observer-reported symptoms of depression (treated as dichotomous response and as a continuous variable and measured using the MADRS^aa^ at week 8); change in functional outcome across 12 months (measured using the SAS^ab^ screener); patients also completed detailed health economic, acceptability, and cognitive functioning measures that will be reported separately	Nil
Jayakumar et al [46], 2021	To assess the effect of an artificial intelligence–enabled patient decision aid that includes education, preference assessment, and personalized outcome estimations (using patient-reported outcome measurements) on decision quality, patient experience, functional outcomes, and process-level outcomes among individuals with advanced knee osteoarthritis considering total knee replacement in comparison with education only	Decision process score of the knee decision quality instrument questions 3.1 to 3.5	Level of shared decision-making (assessed using the CollaboRATE survey); patient satisfaction with the consultation (numerical rating scale); condition-specific symptoms and functional limitations (Knee Injury and Osteoarthritis Outcome Score, Joint Replacement); duration of consultation in minutes; total knee replacement rates (proportion of patients undergoing surgery); treatment concordance (knee decision quality instrument question 1.6)	Nil
Kamba et al [47], 2021	To clarify whether adenoma miss rate could be reduced with the CADe assistance during screening and surveillance colonoscopy	Adenoma miss rate	Polyp miss rate; sessile serrated lesion miss rate; ADR	Further investigation
Luo et al [48], 2021	To explore whether artificial intelligence–assisted colonoscopy could improve the PDR in the actual clinical environment	PDR	Number of polyps detected; the number of diminutive polyps (diameter <6 mm); the number of polyps of each Paris type detected; the number of false positive results	Further investigation
Rafferty et al [49], 2021	To determine whether Heali, a novel artificial intelligence dietary mobile app can improve adherence to the LFD^ac^, IBS^ad^ symptom severity and quality of life outcomes in adults with IBS or IBS-like symptoms over a 4-week period	Adherence to the LFD	IBS symptom severity; quality of life outcomes	Nil
Repici et al [50], 2021	To assess the efficacy of a CADe system in detection of colorectal neoplasias in a nonexpert setting to challenge the CADe impact in a real-life scenario	ADR	Proximal ADR; total number of polyps detected; sessile serrated lesion detection rate; mean number of adenomas per colonoscopy; cecal intubation rate; withdrawal time	Further investigation
Strömblad et al [51], 2021	To assess accuracy and real-world outcome from implementation of a machine learning model that predicts surgical case duration	Accurate prediction of the duration of each scheduled surgery	Effects on patients and systems were measured by start time delay of following cases; time between cases; the time patients spent in presurgical area	Nil
Wu et al [52], 2021	To verify the effectiveness of ENDOANGEL in improving endoscopy quality and pretest its performance in detecting EGC^ae^ in a multicenter randomized controlled trial	Number of blind spots	Performance of ENDOANGEL in predicting early gastric cancer in a clinical setting	Further investigation
Yao et al [53], 2021	To assess whether an ECG-based, artificial intelligence–powered clinical decision support tool enables early diagnosis of low EF^af^, a condition that is underdiagnosed but treatable	Rate of newly diagnosed low EF, defined as EF≤50% within 90 days	Completion of an ECG within 90 days; other findings (eg, valvular heart disease), except low EF present on ECGs	Further investigation
Brown et al [54], 2021	To assess the comparative adenoma miss rate for CADe-assisted colonoscopy when compared with high-definition white light colonoscopy alone	Adenoma miss rate	Polyp miss rate; hyperplastic polyp miss rate; sessile serrated lesion miss rate; ADR; PDR; adenoma per colonoscopy; polyp per colonoscopy; sessile serrated lesion per colonoscopy	Further investigation

^a^ANN: artificial neural network.

^b^CPAP: continuous positive airway pressure.

^c^ICU: intensive care unit.

^d^WATS: wide-area transepithelial sampling.

^e^HGD: high-grade dysplasia.

^f^EAC: esophageal adenocarcinoma.

^g^BE: Barrett’s esophagus.

^h^HPI: hypotension probability indicator.

^i^SBP: systolic blood pressure.

^j^BP: blood pressure.

^k^ASD: autism spectrum disorder.

^l^SRS-II: Social Responsiveness Scale II.

^m^ADR: adenoma detection rate.

^n^PDR: polyp detection rate.

^o^EGD: esophagogastroduodenoscopy

^p^ANSeR: Algorithm for Neonatal Seizure Recognition.

^q^C-EGD: conventional esophagogastroduodenoscopy.

^r^U-TOE: ultrathin transoral endoscopy.

^s^CADe: computer-assisted detection.

^t^PIPA: prostate implant planning algorithm.

^u^BBPS: Boston Bowel Preparation Scale.

^v^ELF-EMF: extremely low frequency and low intensity electromagnetic fields.

^w^NIHSS: National Institutes of Health Stroke Scale.

^x^OHCA: out-of-hospital cardiac arrest.

^y^DA-CPR: dispatcher-assisted cardiopulmonary resuscitation.

^z^QIDS-SR-16: Quick Inventory of Depressive Symptomatology (16-Item) (Self-Report).

^aa^MADRS: Montgomery-Åsberg Depression Rating Scale.

^ab^SAS: Social Adjustment Scale.

^ac^LFD: low fermentable oligo-, di-, mono-saccharides and polyols diet.

^ad^IBS: irritable bowel syndrome.

^ae^ECG: electrocardiogram.

^af^EF: ejection fraction.

### Study Outcomes

Table 3 shows the study results and limitations of each RCT. Of the 39 RCTs, 30 (77%) reported a positive study outcome where AI-assisted interventions outperformed the control arms. Of these 30 studies with positive outcomes, 22 (73%) AI-assisted interventions were biosignal based, and 8 (27%) studies used clinical data–based AI-assisted intervention for clinical outcome improvement. In addition, 21 of these 30 (70%) studies reported positive results of clinically relevant end points. Of these, 18 (86%) led to further investigations, 1 (5%) led to change in treatment, and 2 (9%) reduced the length of hospitalization.

**Table 3:** Study results and limitations.

Author (publication year)	Primary end point result	Secondary end point result	Study outcome	Underpowered	Study conclusion	Limitations
El Solh et al [17], 2019	Time to optimal CPAP^a^ pressure: AI^b^ mean 198.7 (SD 143.8) minutes versus control mean 284.0 (SD 126.5) minutes	Titration failure: AI 16% versus control 36%; drop of residual obstructive apnea–hypopnea events and oxygen desaturations	Positive	No	Maximizing the time to achieve optimal CPAP and in reducing CPAP titration failure	Single center only; possible analysis bias as technologists were not blinded
Shimabukuro et al [18], 2017	AI 10.3 days versus control 13 days	In-hospital mortality: AI 8.96% versus control 21.3%; ICU^c^ length of stay: AI 6.31 days versus control 8.40 days	Positive	No	Significant decrease in the hospital LOS^d^ and in-hospital mortality	Small sample size; heterogenous population; trial was conducted in the 2 ICUs only; metrics were not monitored prospectively during the study because of the likely misrepresentation of such results; false positive rate, sensitivity, and prediction rate may be affected as clinicians may have initiated treatment before severe sepsis onset owing to advanced notice from the predictive algorithm; the use of overall metrics, LOS, and in-hospital mortality for all comers may underestimate the impact of the intervention on outcomes for patients with sepsis; potential for competing risks in the selected end points, mortality may shorten a patient’s LOS; this study was patient-outcome oriented
Labovitz et al [6], 2017	Mean (SD) cumulative adherence based on pill count was 97.2 (4.4%) for the AI platform group and 90.6% (5.8%) for the control group. Plasma drug concentration levels indicated that adherence was 100% (15/15) and 50% (6/12) in the intervention and control groups, respectively	Nil	Positive	Unknown	Real-time monitoring has the potential to increase adherence and change behavior, particularly in patients on direct oral anticoagulant therapy	Not mentioned
Gracey et al [19], 2018	Likelihood of being adherent: AI>control, 6.11%; likelihood of being adherent: AI>traditional, 7.8%; no significant difference in likelihood of being adherent	Nil	Positive	Unknown	Using AI to target interventions can increase the effectiveness of medication adherence intervention programs	Not mentioned
Liu et al [20], 2018	AI versus control: no significant difference	Economic burden of disease—AI versus control (all): no significant difference; economic burden of disease—AI: 46,006 (SD 40,831) yuan (US $6901 [SD 6125]) versus control (in surgical dept): 64,192 (SD 67,968) yuan (US $9629 [SD 10195])); benefit-cost ratio of AI: 1.15; net present value of benefit-cost of AI: 5792 yuan; direct medical costs—AI: 43,467 (SD 39.716) versus control: 61,205 (SD 66,576) yuan	Negative	Unknown	A clinical decision support system based on the graph-based machine learning algorithms changed the antihypertensive prescriptions and reduced the medical expense among patients with hypertension	Not mentioned
Vennalaganti et al [21], 2018	HGD^e^ or EAC^f^ detection—WATS^g^ alone: 29 versus control alone: 7; AI (alone)>control (alone) 4.2 times	Neoplasia detection rates: not mentioned; average time required for WATS; additional time required for WATS: 11 minutes 26 seconds versus control: 6 minutes 55 seconds	Positive	Unknown	WATS increases the detection of HGD and EAC in a high-risk BE^h^ surveillance population when used as an adjunct to biopsy sampling compared with biopsy sampling alone	Single center research only; potential of population bias as study population (20%) was enriched with patients with BE with a known history of dysplasia or referred for endoscopic therapy; no long-term follow-up
Biester et al [22], 2019	AI: 66.6% versus control: 59.9%	Percentage <60 mg/dL—AI: 0.64% versus control: 0.38%; percentage <70 mg/dL—AI: 2.31% versus control: 1.45%; percentage >180 mg/dL—AI: 28.32% versus control: 36.43%; percentage >240 mg/dL—AI: 8.53% versus control: 8.71%; Mean —AI: median (IQR) 153.11 (142.33-174.81) versus control: 163.84 (150.17-186.54); SD—AI: median (IQR) 52.71 (44.75-66.39) versus control: 54.95 (46.19-69.19)	Positive	No	The MD-Logic system was safe and associated with better glycemic control than SAP^i^ therapy for day and night use. The absence of remote monitoring did not lead to safety signals in adapting basal rates nor in administration of automated bolus corrections	High rate of communication errors between the tablet computer running the algorithm and the insulin pump
Pouska et al [23], 2019	No significant difference in number of hypotension events between 2 groups (4/20 vs 2/20)	AI: 10 versus control: 4	Negative	Unknown	On the basis of our data, it seems that the inclusion of HPI^j^ into a goal-directed treatment strategy could lower the incidence of hypotension within maintenance phase of anesthesia	Not mentioned
Lin et al [24], 2019	Accuracy—AI: 87.4% versus control: 99.1%	No significant difference in evaluation of the disease severity between AI and control; AI: 2.79 minutes versus control: 8.53 minutes; rating of overall satisfaction—AI: mean 3.47 (SD 0.501) versus control: mean 3.38 (0.554)	Negative	No	CC-Cruiser exhibited less accuracy compared with senior human consultants in diagnosing childhood cataracts and making treatment decisions, but it has the capacity to assist human physicians in clinical practice in its current state	Patients without symptoms were less willing to participate in; patients with slightly opaque lens may have missed; CC-Cruiser provided treatment suggestions without considering the patients’ general conditions; lack of internet accessibility limited the implementation of CC-Cruiser in low-income areas; possibly sufficient statistic power because cluster RCT^k^ was adopted in trial, whereas RCT was used in sample size calculation
Kamdar et al [25], 2019	Difference of BPI^l^ between AI and control: ß=−.09	Difference of BQ-II^m^ between AI and control: ß=−.037; difference of General Anxiety Disorder-7 between AI and control: ß=.21; AI: 4 versus control: 20	Positive	Unknown	AI significantly decreases pain scores and pain-related hospitalizations in patients with cancer-related pain	Not mentioned
Persell et al [26], 2020	AI: mean systolic blood pressure (SD) 132.3 (15.0) mm Hg versus control: 135 (13.9) mm Hg	Significant improvement in self-reported medication adherence in AI group than control; no significant difference between home monitoring and self-management practices; AI group has 26.7 minutes per week (−5.4 to 58.8) more than control group in self-reported physical activity	Negative	Unknown	Adults with hypertension randomized to a coaching app plus home monitor had similar SBP^n^ compared with controls receiving a tracking app and home monitor	Blinding to participants and research staff is impossible; some outcomes were self-reported; not specifically select participants who were likely to use a health-coaching app; small sample size; the app used in the study was a beta version; the AI and machine learning technology used here in this app gains information with larger numbers of users contributing data; cannot exclude the possibility that some patients may have well-controlled hypertension; limited generalizability because only iOS device users were recruited
Voss et al [27], 2019	SRS-II^o^ showed large, not significant, positive mean changes in treatment participants; the VABS-II^p^ socialization subscale score significantly increased between the start and end of the intervention in treatment-to-control comparisons	Moderator analyses showed a moderation effect for girls showing greater improvement; no significant changes from intake to posttest 1 were observed on Child Behaviour Checklist; the VABS-II adaptive composite score showed slightly greater improvement in younger participants	Positive	Yes	This study underscores the potential of digital home therapy to augment the standard of care	According to the poststudy empirical variance, this study may be underpowered by a factor of 2; low treatment adherence; bias in recruitment of participants; bias owing to the inherent demographic and behavioral heterogeneity of patients; second posttest appointments were not available for control participants before crossing over into treatment
Wang et al [28], 2019	AI: 29.1% versus control: 20.3%	AI: 0.45 versus control: 0.29 (OR^q^ 1.995, 95% CI 1.532-2.544); AI: 0.97 versus control: 0.51; AI: 0.53 versus control: 0.31; false positive rate of AI: 0.075 per colonoscopy; false negative rate of AI: not mentioned	Positive	No	In a low prevalent ADR^r^ population, an automatic polyp detection system during colonoscopy resulted in a significant increase in the number of diminutive adenomas detected as well as an increase in the rate of hyperplastic polyps	Endoscopists were not blinded; lack of external validity; despite low false positive rates, potential distraction during the procedure could also be caused; fatigue level of participating endoscopists were not controlled; inadequate sample size of colonoscopies performed by junior endoscopists; only Olympus colonoscopy equipment was used
Wu et al [29], 2019	AI: 5.86% versus control: 22.46%	AI: 5.03 minutes versus control: 4.24 minutes; AI: 71.87% versus control: 79.14%; AI: 90.64% versus control: 79.14%; AI: 92.91% versus control: 79.14%; percentage of patients being ignored in majority gastric sites were significantly lower than control	Positive	No	WISENSE greatly reduced blind spot rate, increased inspection time, and improved the completeness of photo documentation	Only Olympus and Fujifilm endoscopes were used in this trial; the withdrawal time in this trial was generally less than recommended 7 minutes of EGD^s^ in the guideline
Pavel et al [30], 2020	Diagnostic accuracy (sensitivity, specificity, and false detection rate) for recognition of a neonate with seizures were not significantly different between the 2 groups; sensitivity of seizure hours—AI: 66% versus control: 45.3%; false detection rate of seizure hours was not mentioned	No significant differences found in seizure characteristics; AI: 37.5% versus control: 31.6%; difference 5.9%	Negative	No	In conclusion, this clinical investigation was the first to assess the performance of a machine learning algorithm for neonatal seizure detection in real time and in the real-world setting of busy neonatal ICUs throughout Europe	Excluded seizures with a duration of <30 seconds from both groups; analysis was done using seizure hour instead of looking at each individual seizure
Alfonsi et al [31], 2020	Absolute error at 3-month follow-up—AI: 27.45% (10.90%) versus control: 38.00% (14.74%); error>10 g at 3-month follow-up—AI: 21.43% (16.82%) versus control: 32.27% (16.31%)	No significant difference between groups A and B in terms of accuracy, procedural safety, time for trocar placement, and patient radiation exposure (dose area product and fluoroscopy time)	Positive	No	The data suggest that use of iSpy is associated with improved carbohydrate counting and that usability and acceptability of the app is quite positive	Single tertiary pediatric center only; the number of foods recognized by iSpy is not all encompassing; detailed information about other factors that can influence care such as education level, socioeconomic status data, family dynamics, or details of treatment regimen were not acquired; text reminders to the control participants was not provided
Auloge et al [32], 2020	Group A technical feasibility was 100% with successful segmentation and generation of safe or accurate trajectory in all cases	No significant difference in accuracy; no complications or unintended effects were observed in either group—AI: mean 642 (SD 210) seconds, range 300-963 versus control: mean 336 (SD 60) seconds, range 240-438; DAP^t^—AI: mean 182.6 (SD 106.7) mGy cm^2^, range 27-355 versus control: mean 367.8 (SD 184.7) mGy cm^2^, range 115-644; fluoroscopy time—AI: 5.2 (SD 2.6) seconds, range 1.6-8.7 versus control: mean 10.4 (SD 4.1) seconds, range 4.2-17.9	Positive	Yes	Augmented reality or AI–guided percutaneous vertebroplasty appears feasible, accurate and safe and facilitates lower patient radiation exposure compared with standard fluoroscopic guidance	Small sample size; surgeon bias due to inherent nonblinding; lack of power to assess differences in vertebroplasty complication rates; accuracy of final trocar position was estimated on augmented fluoroscopic images rather than CBCT^u^; no clinical follow-up is presented
Avari et al [33], 2020	AI: 62.5% (52.1%-67.8%) versus control: 58.4% (49.6%-64.3%)	No significant difference for percentage of time in euglycemia, hypoglycemia, and hyperglycemia; no episode of serious hypoglycemia; no episodes of hypoglycemia within 5 hours postprandially; case of severe hypoglycemia; AI: 0 versus control: 1; no significant difference in glycemic risk	Negative	Yes	The PEPPER system was safe but did not change glycemic outcomes compared with control	The potential need for additional time required for the adaptive insulin recommender system to be effective; the algorithm is likely to be most beneficial to individuals maintaining regular work patterns rather than shift workers; the algorithm only adapts for bolus insulin and assumes that the basal insulin has been optimized; the system is dependent on meal scenarios where the user has not ingested a significant snack or taken an insulin bolus correction within 5 hours of a meal for revision
Chen et al [34], 2020	Sedated C-EGD versus unsedated U-TOE^v^ versus unsedated C-EGD: 3.42% (0.89/26) versus 21.77% (5.66/26) versus 31.23% (8.12/26), respectively	Blind spot rate of Sedated C-EGD—AI: 3.42 versus control: 22.46; blind spot rate of unsedated U-TOE—AI: 21.77 versus control: 29.92; blind spot rate of unsedated C-EGD—AI: 31.23 versus control: 42.46; the average accuracy, sensitivity, and specificity of EN-DOANGEL in sedated C-EGD were 88.3%, 92.6%, and 90.2%, respectively; in unsedated U-TOE, were 91.3%, 84.5%, and 90.1%, respectively; and in unsedated C-EGD, were 87.8%,8 2.8%, and 87.8%, respectively	Positive	No	In summary, our study showed that the number of blind spots in conventional sedated EGD was the lowest compared with unseated U-TOE and unsedated EGD, and the addition of ENDOANGEL had a maximal effect on unsedated C-EGD	Single-center study; endoscopist were not blinded
Gong et al [35], 2020	ITT^w^—AI: 16% (58/355) versus control: 8% (27/349); PP^x^—AI: 17% (54/224) versus control: 8% (26/318)	ITT diminutive—AI: 46 (13%) versus control: 25 (7%); ITT small—AI: 4 (1%) versus control: 1 (<1%); ITT large—AI: 10 (3%) versus control: 1 (<1%); no significant differences were found comparing adenoma locations: ITT—AI: 47% (166/355) versus control: 34% (118/349); ITT diminutive—AI: 158 (45%) versus control: 114 (33%); ITT small—AI: 9 (3%) versus control: 7 (2%); ITT large—AI: 11 (3%) versus control: 3 (1%); significant different was only found in sigmoid colon—AI: 79 (22%) versus control: 48 (14%); AI: 0.18 versus control: 0.08; AI: 1.17 versus control: 0.68; AI: 6.38 minutes versus control: 4.76 minutes; no adverse and serious adverse events	Positive	No	In conclusion, the ENDOANGEL system is a quality improving system for colonoscopy that uses computer vision, real-time monitoring of withdrawal speed, and timing of colonoscopy intubation and withdrawal and provides reminders to endoscopists of blind spots, in addition to live tracking previously seen frames during colonoscopy	AI was validated at 1 center only; the withdrawal speed was artificially divided into safe, alarm, and dangerous by assessing videos from Renmin Hospital of Wuhan University; the difference between assisted and unassisted colonoscopy for adenomas of 6-9 mm was not significant, which could be attributable to small numbers
Liu et al [36], 2020	AI: 29.01% versus control: 20.91%	AI: 47.07% versus control: 33.25%; AI: 1.07 versus control: 0.51; AI: 0.48 versus control: 0.29	Positive	No	In conclusion, real-time visual alarms provided by a high-performance CADe^y^ system embedded into the primary colonoscopy monitor, with nearly unnoticeable latency, have been shown to cause a significant improvement in ADR because of an increased detection of diminutive adenomas without increasing physician fatigue level during colonoscopy	Open-labeled study; the fatigue score was subjective and susceptible to factors other than the visual alarms; whether a polyp was first detected by CADe before the endoscopist was based on the operating endoscopist’s own judgment; the fact that the CADe system detected a polyp before the endoscopists does not necessarily mean that the endoscopists would have missed that lesion
Nicolae et al [37], 2020	No significant difference in CTV^z^ V_100_, CTV D_90_, and Rectum V_100_ at 1-month postoperative follow-up	AI: mean 2.38 (SD 0.96) minutes versus control: mean 43.13 (SD 58.70) minutes; no significant difference in need and extent of modifications	Positive	Yes	A machine learning–based planning workflow for prostate LDR^aa^ brachytherapy has the potential to offer significant time savings and operational efficiencies, while producing noninferior postoperative dosimetry to that of expert, conventional treatment planners	Single-center study; examining only preoperatively planned cases
Repici et al [38], 2020	AI: 54.8% versus control: 40.4%	AI: 123 versus control: 97; AI: 353 out of 262 patients versus control: 243 out of 198 patients; AI: 7% versus control: 5.2%; AI: 1.07 versus control: 0.71; AI: 95.6% versus control: 98.5%; withdrawal time: not mentioned	Positive	No	In a multicenter, randomized trial, we found that including CADe in real-time colonoscopy significantly increases ADR and adenomas detected per colonoscopy without increasing withdrawal time	Psychological bias could not be excluded; the equivalence in withdrawal time excludes a somewhat reduced degree of mucosal exposure in the control arm; low detectors and inexperienced or nongastroenterologist endoscopists were not involved in this study
Su et al [39], 2020	AI: 28.90% versus control: 16.51% (OR 2.055, 95% CI 1.397-3.024; *P*<.001)	AI: 38.31% versus control: 25.40%; AI: 0.367 versus control: 0.178; AI: 0.575 versus control: 0.305; AI: mean 7.03 (SD 1.01) minutes versus control: mean 5.68 (SD 1.26) minutes; AI: 87.34% versus control: 80.63%	Positive	No	In summary, AQCS^ab^, an automatic quality control system, could be used in real time for timing, supervising withdrawal stability, evaluating BBPS^ac^, and detecting polyp	Single endoscopic center; some false prompts occurred with the AQCS; fatigue level of participating physicians was not controlled; used 4 intraprocedural quality metrics to form the AQCS, without performing preliminary testing to evaluate whether just 2 or 3 or 4 of these metrics had the same quality improvement; did not test the sole effect of colonoscopy stability; the DCNNs^ad^ were trained only on images obtained from a Pentax imaging system
Wang et al [40], 2020	AI: 13.89% versus control: 40%	AI: 12.98% versus control: 45.90%; no statistical differences in the miss rate of advanced adenomas and sessile serrated adenoma or polyps; no significant difference in patient miss rate; no significant difference in ADR for the first pass; no significant difference in adenoma per colonoscopy; no significant difference in polyp per colonoscopy	Positive	No	The results from this study suggest a significantly lower AMR^ae^ when a CADe technology is used compared with routine white light colonoscopy. The detection of diminutive and small adenomas with nonadvanced histology and nonpedunculated shape could be effectively improved by CADe colonoscopy	AMR obtained in the tandem study cannot reflect the absolute miss rate; subjective bias in open-labeled trial; tandem colonoscopy in each patient was performed by the same endoscopist; study population was not restricted to screening-only participants according to guidelines; only skilled endoscopists were allowed to participate in this study; subjected bias may be introduced as the judgments made by the panel of 3 experts who reviewed the video record were not a gold standard as pathology
Wang et al [41], 2020	AI: 165 (34%) versus sham control: 132 (28%)	AI: 252 (52%) versus sham control: 176 (37%); AI: 1.04 versus sham control: 0.64; AI: 0.58 versus sham control: 0.38	Positive	No	The CADe system is a safe and effective method to increase ADR during colonoscopy	Potential bias in the presence of a second senior endoscopist; bias in patient recruitment; the actual alert numbers of the sham system should have been measured in the trial to show equivalence
Wijnberge et al [42], 2020	AI: median 0.10, IQR 0.01-0.43 mm Hg versus control: median 0.44, IQR 0.23-0.72 mm Hg	AI: 3.00, IQR 1.00-8.00 versus control: 8.00, IQR 3.50-12.00; AI: 8.00, IQR 1.33-26.00 minutes versus control: 32.67, IQR 11.50-59.67 minutes; AI: 2.8%, IQR 0.8%-6.6% versus control: 5.6%, IQR 3%-9.4%; AI: 2.0 (0.0 to 3.0) versus control: 0.0 (−1.0 to 0.0); AI: 4.0 (0.0 to 10.7) minutes versus control: −0.7 (−4.3 to 0.7) minutes; AI: 1.5% (0.0 to 3.3) versus control: −0.2% (−1.4 to 0.3)	Positive	No	In this single-center preliminary study of patients undergoing elective noncardiac surgery, the use of a machine learning–derived early warning system compared with standard care resulted in less intraoperative hypotension. Further research with larger study populations in diverse settings is needed to understand the effect on additional patient outcomes and to fully assess safety and generalizability	Single center only; small sample size; patient may have their own personal minimal MAP to be maintained during surgery; depth of anesthesia was not measured; the early warning system is validated only for invasive continuous blood pressure monitoring; an observer being present in the operating room may have influenced protocol adherence
Weisinger et al [43], 2021	Fugl-Meyer Assessment-Upper Extremity: week 4—AI: mean 23.2 (SD 3.91) versus control: mean 9.9 (SD 3.2); week 8—AI: mean 31.5 (SD 2.97) versus control: mean 23.1 (SD 4.99)	AI: 2.5 (0.18) points versus control: 1.3 (0.16) points; significance improved: Action Research Arm Test-Pinch subscale; significance improved: Box and Block Test; significance improved: NIHSS^ae^	Positive	Unknown	BQ treatment significantly improves upper extremity motor function in a population with subacute ischemic stroke across multiple clinical metrics. Further studies are planned and ongoing with larger study populations and in related indications	Nil
Blomberg et al [44], 2021	AI: 93.1% (296/318) versus control: 90.5% (304/336)	AI: 1.72 (1.52) minutes versus control: 1.70 (1.63) minutes; AI: 64.8% versus control: 61.9%	Negative	Yes	This randomized clinical trial did not find any significant improvement in dispatchers’ ability to recognize cardiac arrest when supported by machine learning even though AI did surpass human recognition	Not 100% compliance with the machine learning model; the servers analyzing the phone calls had downtime, because the server was underdimensioned
Browning et al [45], 2021	QIDS-SR-16^af^ at week 8—AI: 55.9% versus control: 51.8	Generalized Anxiety Disorder Assessment, 7 item version (week 8)—AI: −5.44 versus control: −6.12	Negative	Yes	Use of a predictive algorithm to guide antidepressant treatment improves symptoms of anxiety and functional outcomes provides initial support for the use of personalized medicine approaches in the treatment of depression	The accuracy of the predictive algorithm was modest at 57.5%; effectiveness was focused rather than efficacy, requesting but not requiring clinicians to alter treatment in response to a prediction of nonresponse; randomization occurred at the level of the patient rather than the site, and thus, the treatment as usual arm may have been influenced by behavior learned in the active arm
Jayakumar et al [46], 2021	AI: mean 68.9 (SD 19.8) versus control: mean 48.8 (SD 14.5)	CollaboRATE median—AI: 8 of 69 versus control: 28 of 60; number of patient-rated satisfaction scores lower than the median value of 10—AI: 9 of 69 versus control: 19 of 60; no significant difference in duration of consultation in minutes; no significant difference in TKR^ag^ rates and treatment concordance	Positive	No	In this randomized clinical trial, an AI-enabled decision aid significantly improved decision quality, level of shared decision-making, satisfaction, and physical limitations without significantly impacting consultation times, TKR rates, or treatment concordance in patients with knee osteoarthritis considering TKR. Decision aids using a personalized, data-driven approach can enhance shared decision-making in the management of knee osteoarthritis	Single-center study; surgeons were not masked; we did not assess the effect of the decision aid on patient knowledge; the typical course of a formal osteoarthritis in-clinic diagnosis possesses a general limitation in limiting the time frame over which the tool may be applied
Kamba et al [47], 2021	AI first: 13.8% versus control first: 35.7%	AI first: 14.2% versus control first: 40.6%; AI first: 13% versus control first: 38.5%; AI first: 64.5% versus control first: 53.6%	Positive	No	The reduction of AMR by assisting with CADe based on deep learning in a multicenter randomized controlled trial	Nil
Luo et al [48], 2021	AI: 38.7% versus control: 34%	The number of polyps detected in the control group and the research group was 80 and 105, respectively; AI: 91 versus control: 69; polyp type 0-IIa—AI: 87 versus control: 61; polyp type 0-Is—AI: 5 versus control: 8; polyp type 0-Ip—AI: 13 versus control: 11; 52 false positive result in AI group; in average, 0.35 false positive per colonoscopy	Positive	No	This study shows that an AI system based on deep learning and its real-time performance led to significant increases in colorectal PDR^ah^	Single center study; small sample size; AI has different effects on improving the PDR among different physicians; ADR was not compared between 2 groups in this trial
Rafferty et al [49], 2021	IBS^ai^ symptom score—AI: −170 versus control: −138; quality of life score—AI: 31.1 versus control: 11.8	No significant difference; AI: 8.3 (4.4-13.1) versus control: 10.4 (7.4-14.0)	Negative	Yes	Results showed that the Heali app was able to significantly increase quality of life outcomes in IBS participants over a 30-day intervention period	Small sample size; self-reporting bias in survey may resulted owing to lack of blinding; stratification was not done; participants were not randomized to groups until study day 10, which was after the collection of baseline data; although anthropometric measures (bodyweight and height) were collected at baseline, they were not collected at the end of the trial, and it is possible that changes in body weight influenced the outcome variables; adherence may be affected by social impacts of the COVID-19 pandemic
Repici et al [50], 2021	AI: 176/330, 53.3% versus control: 146/330, 44.2%	AI: 41.5% versus control: 36.1%; AI: 1.98 (range 0-15) versus control: 1.61 (range: 0-17); AI: 3.3% versus control: 5.2%; AI: 1.26 (SD 1.82) versus control: 1.04 (SD 1.75); 100% in both groups after excluding patients with inadequate bowel preparation; AI: mean 815 (SD 1.6) versus control: mean 7.98 (SD 1.5)	Positive	No	CADe in real-time colonoscopy significantly increases ADR and adenomas detected per colonoscopy in a nonexpert setting	No comparison of AI assistance with alternative educational interventions among inexpert endoscopists; this study design was not fit to assess the sensitivity or specificity of the device; no power calculations were done for any of our secondary outcomes
Strömblad et al [51], 2021	Mean absolute error—AI: 49.5 minutes {66} versus control: 59.3 minutes {72}	Mean patient wait time: overall—AI: 16.3 minutes versus control: 49.4 minutes (67.1% improvement); turnover time: overall—AI: 69.1 minutes versus control: 70.6 minutes (2% improvement); patient time in facility—AI: 148.1 versus control: 173.3 (14.5% improvement)	Positive	No	Implementing machine learning–generated predictions for surgical case durations may improve case duration accuracy, presurgical resource use, and patient wait time, without increasing surgeon wait time between cases	Small sample size; prediction accuracy may be affected if the submitted procedure codes deviate significantly from the procedures that are performed; a less common occurrence were multipanel cases in which multiple surgeons from different services operated on the same patient during the same case; there was no stratification by days
Wu et al [52], 2021	AI: 5.38 (SD 4.32) versus control: 9.82	AI: 5.40 (SD 3.82) minutes versus control: 4.38 (SD 3.91) minutes; the median percentage of patients with blind spots at each site—AI: 21% versus control: 38.9%; per-lesion accuracy: 84.7%; sensitivity: 100%; specificity: 84.3%	Positive	No	ENDOANGEL was an effective and robust system to improve the quality of EGD and has the potential to detect electrocardiogram in real time	We only conducted a feasibility analysis on real-time detection of gastric cancer based on deep learning in a clinical setting; the enrolled patients were not followed up for a long time; statisticians were not blinded
Yao et al [53], 2021	In overall cohort—AI: 2.1% versus control 1.6%; among 1356 patients who had a positive result—AI: 19.5% versus control: 14.5%	No significant between AI and control on disease discovery	Positive	No	An AI algorithm run on existing electrocardiograms enabled the early diagnosis of low ejection fraction in a large cohort of patients managed in routine primary care practices. Because electrocardiography is a low-cost test that is frequently performed for a variety of purposes, the algorithm could potentially improve early diagnosis and treatment of a condition that is often asymptomatic but has effective treatments and thus reduce the disease burden in broad populations	Echocardiogram may not be ordered by clinician as nearly all the patients had insurance coverage; study was not designed to determine the long-term clinical impact; for example, heart failure hospitalizations and mortality
Brown et al [54], 2021	AMR—CADe first: 20.12% versus HDWL^aj^ first: 31.25%	PDR—CADe first: 20.7% versus HDWL first: 33.71%; HPMR^ak^—no significant difference in the hyperplastic polyp miss rate; SSLMR^al^—CADe first: 7.140% versus HDWL first: 42.11%; no statistically significant difference in ADR during first pass, second pass, and whole process; no statistically significant difference in PDR during first pass, second pass, and whole process; adenoma per colonoscopy during first pass—CADe first: 1.19 versus HDWL first: 0.90; no statistically significant difference during second pass and whole process; polyp per colonoscopy during first pass—CADe first: 2.0 versus HDWL first: 1.59; polyp per colonoscopy during second pass—CADe first: 0.52 versus HDWL first: 0.81; no statistically significant difference during whole process; SSLPC^am^ during second pass—CADe first: 0.01 versus HDWL first: 0.07; no statistically significant difference during first pass whole process	Positive	No	This study showed a decrease in AMR with the use of a deep learning CADe system when compared with HDWL colonoscopy alone and a decrease in polyp and sessile serrated lesion miss rates and an increase in first-pass adenomas per colonoscopy	Not powered to detect a difference in ADR; the tandem colonoscopy design limited in terms of generalizability to the real-world clinical setting; only included experienced endoscopists with a high baseline ADR at US academic medical centers; used a second monitor adjacent to the primary endoscopy monitor

^a^CPAP: continuous positive airway pressure.

^b^AI: artificial intelligence.

^c^ICU: intensive care unit.

^d^LOS: length of stay.

^e^HGD: high-grade dysplasia.

^f^EAC: esophageal adenocarcinoma.

^g^WATS: wide-area transepithelial sampling.

^h^BE: Barrett's esophagus.

^i^SAP: sensor-augmented pump.

^j^HPI: hypotension probability indicator.

^k^RCT: randomized controlled trial.

^l^BPI: Brief Pain Inventory.

^m^BQ-II: Barriers Questionnaire II.

^n^SBP: systolic blood pressure.

^o^SRS-II: Social Responsiveness Scale II.

^p^VABS-II: Vineland Adaptive Behavioural Scales, Second edition.

^q^OR: odds ratio.

^r^ADR: adenoma detection rate.

^s^EGD: esophagogastroduodenoscopy.

^t^DAP: dose–area product.

^u^CBCT: cone-beam computed tomography.

^v^U-TOE: ultrathin transoral endoscopy.

^w^ITT: intention to treat.

^x^PP: per protocol.

^y^CADe: computer-assisted detection.

^z^CTV: clinical target volume.

^aa^LDR: low-dose rate.

^ab^AQCS: automatic quality control system.

^ac^BBPS: Boston bowel preparation scale.

^ad^DCNN: deep convolutional neural networks.

^ae^AMR: adenoma miss rate.

^af^QIDS-SR-16: Quick Inventory of Depressive Symptomatology (16-Item) (Self-Report).

^ag^TKR: total knee replacement.

^ah^PDR: polyp detection rate.

^ai^IBS: irritable bowel syndrome.

^aj^HDWL: high-definition white light.

^ak^HPMR: Hyperplastic polyp miss rate.

^al^SSLMR: sessile serrated lesion miss rate.

^am^SSLPC: sessile serrated lesion per colonoscopy.

### Study Limitations

The most common limitations listed by the authors among these studies were single-center study design (22/39, 56%) and small sample size (n<1000; 33/39, 85%). This limits the generalizability and statistical power of the AI-assisted tools in different studies. There were 7 studies which were underpowered because of small sample size. Of these, 5 (71%) studies included <100 participants. Another common limitation is the open-label design (15/39, 38%).

### Assessment of Risk of Bias

Detailed assessment results of the risk of bias using the second version of the Cochrane risk-of-bias tool for randomized trials are reported in Table 4. On the basis of the overall risk-of-bias assessment, 20% (8/39) of the trials had a low risk of bias, 31% (12/39) trials had some concerns, and 49% (19/39) had a high risk of bias. Missing outcome data and outcome measurements were the most common risk factors.

**Table 4:** Quality assessment outcome based on the second version of the Cochrane risk-of-bias tool for randomized trials.

Author (publication year)	Randomization process	Deviations from intended interventions	Missing outcome data	Measurement of the outcome	Selection of the reported result	Overall bias
El Solh et al [17], 2009	Low	Low	Low	Low	Low	Low
Shimabukuro et al [18], 2017	Low	Low	Low	Low	Low	Low
Labovitz et al [6], 2017	Some concerns	Low	Low	Low	Low	Some concerns
Gracey et al [19], 2018	Some concerns	High	High	High	High	High
Liu et al [20], 2018	Some concerns	Some concerns	High	High	Some concerns	High
Vennalaganti et al [21], 2018	Some concerns	Low	Low	High	Low	High
Biester et al [22], 2019	Some concerns	Some concerns	High	Low	High	High
Pouska et al [23], 2019	Some concerns	High	Low	Low	Low	High
Lin et al [24], 2019	Low	Low	Low	Low	Low	Low
Kamdar et al [25], 2019	Some concerns	Some concerns	High	High	Some concerns	High
Persell et al [26], 2020	Low	High	Low	High	Low	High
Voss et al [27], 2019	High	Some concerns	Low	High	Low	High
Wang et al [28], 2019	Some concerns	Low	Low	Low	Low	Some concerns
Wu et al [29], 2019	Some concerns	Low	Low	Low	Low	Some concerns
Pavel et al [30], 2020	Some concerns	Low	Low	Low	Low	Some concerns
Alfonsi et al [31], 2020	Some concerns	Low	Low	High	Low	High
Auloge et al [32], 2020	Some concerns	Some concerns	Low	Low	Low	Some concerns
Avari et al [33], 2020	Low	Some concerns	Low	Some concerns	Some concerns	Some concerns
Chen et al [34], 2020	Some concerns	Low	Low	Low	Low	Some concerns
Gong et al [35], 2020	Low	Low	Low	Low	Low	Low
Liu et al [36], 2020	Some concerns	Low	Low	Some concerns	Low	Some concerns
Nicolae et al [37], 2020	Some concerns	Some concerns	High	Low	Low	High
Repici et al [38], 2020	Some concerns	Low	Low	Low	Low	Some concerns
Su et al [39], 2020	Low	Low	Low	Low	Low	Low
Wang et al [40], 2020	Low	Low	Low	High	Low	High
Wang et al [41], 2020	Low	Low	Low	Some concerns	Low	Some concerns
Wijnberge et al [42], 2020	Some concerns	Low	Low	Low	Low	Some concerns
Weisinger et al [43], 2021	Some concerns	Low	High	High	Low	High
Blomberg et al [44], 2021	Low	Low	High	Low	Low	High
Browning et al [45], 2021	Low	Low	Low	Low	Low	Low
Jayakumar et al [46], 2021	Some concerns	Low	Low	High	Low	High
Kamba et al [47], 2021	Some concerns	High	High	Some concerns	Low	High
Luo et al [48], 2021	Some concerns	Low	Low	Low	Low	Some concerns
Rafferty et al [49], 2021	High	Low	Low	High	Low	High
Repici et al [50], 2021	Some concerns	Some concerns	High	High	Low	High
Strömblad et al [51], 2021	Low	Low	Low	Low	Low	Low
Wu et al [52], 2021	Low	Low	Low	Low	Low	Low
Yao et al [53], 2021	Some concerns	High	High	High	Low	High
Brown et al [54], 2021	Low	Low	Low	High	Low	High

## Discussion

### Principal Findings

Despite the plethora of claims for the benefits of AI in enhancing clinical outcomes, there is a paucity of robust evidence. In this systematic review, we identified only a handful of RCTs comparing AI-assisted tools with standard-of-care management in various medical conditions. Among these RCTs, two-thirds demonstrated improved primary or secondary end points compared with the standard-of-care management. However, not all of these end points are clinically relevant, that is, leading to a change in the management plan, improving the treatment results, shortening or avoiding hospital admissions, or reducing mortality. Although we acknowledge that our definition of a *clinically relevant end point* may be relatively narrow, we believe that the absence of such end points in the RCTs shows a clear deficit in the available evidence.

As expected, most of these studies came from economically advanced, industrialized countries, which constituted two-thirds of the RCTs included in this systematic review. China, as a single nation, accounted for one-third of the RCTs. China’s research in this area is empowered by immense amount of resources invested in AI or machine learning (ML), internet, its vast patient population, and the availability of a nationwide electronic health record for hospitalized patients [55]. The geographical distribution of these studies is important as AI or ML relies on data fed to the system. Differences in genomic, metagenomic, and even environmental factors may influence disease patterns and the presentation of diseases. Therefore, it is desirable to develop an AI or ML tool based on data collected from different ethnic groups and tested in individual regions to prove its efficacy. There is only one AI-assisted tool, EndoScreener, which uses different ethnic groups in its development and validation. It was originally developed and trained using a different data set of endoscopic images including an open-source database of endoscopic images from Spain. Subsequently, the tool was validated in 4 prospective RCTs in China and had been recently validated in a multicenter RCT in the United States, proving its effectiveness. Future studies should focus on validation of AI-assisted tools across different ethnic groups and patient populations to ensure generalizability.

More biosignal-based AI-assisted tools have been studied than clinical data–based tools in RCTs. The most widely used were endoscopic images detecting adenoma during colonoscopy. The adenoma detection algorithm appears to be easier for cross-compatibility because of the distinct difference in appearance between adenoma (or polyp) and normal mucosa [56]. There were a total of 5 different AI-assisted adenoma or polyp detection systems tested in 9 separate RCTs, all of which successfully assisted endoscopists to detect more adenomas or polyps during colonoscopy. However, only one study successfully showed that the AI-assisted adenoma detection system could improve adenoma detection of all sizes. Other studies could only show improvement in diminutive adenoma (<5 mm) or small adenoma (<10 mm) detection [39]. Advanced adenoma or colorectal cancer detection was not improved by the AI-assisted adenoma detection system used in these studies. The US Multi-Society Task Force on Colorectal Cancer [57] suggested that patients with 1 to 2 nonadvanced adenoma sized <10 mm are at low risk and could have their surveillance colonoscopy in 7 to 10 years. The value of improvement in diminutive or small adenoma detection is uncertain. Among all studies, there was only one reported long-term outcome, that is, in-hospital mortality. Future studies should emphasize on the impact of AI-assisted tools on the long-term clinical end points.

Classical prediction models are typically clinical risk scores derived from regression-based statistical models, which could be considered an ML model that has been modified for clinical use. Ideally, RCTs should be designed with a control arm (usual clinical care), a “standard-of-care” clinical risk score arm, and a novel AI-assisted tool arm. However, given the expense and effort of a clinical trial, the SPIRIT-AI (Standard Protocol Items: Recommendations for Interventional Trials-Artificial Intelligence) extension guidelines clearly state the importance of pre-existing evidence for AI intervention, with evidence that the AI-assisted tool produces better performance compared with the standard of care [58]. Although none of the RCTs found in this systematic review used a multi-arm design, there have been well-designed studies where more “complex” ML approaches have outperformed regression-derived clinical risk scores on external validation [7,59,60].

### Conclusions and Recommendations

The findings of this systematic review are by no means to discourage the use of AI in medicine. AI or ML can detect signals in an immense data pool to develop algorithms for clinical decision. Unlike humans, AI or ML can process enormous quantities of data, perform consistently, and constantly improve its performance by learning from new data. However, for AI or ML to be implemented in daily clinical practice, assisting clinicians in making important decisions, proof-of-concept evidence is not sufficient. AI-assisted tools must demonstrate unequivocal improvement in clinically relevant outcomes in properly designed randomized controlled clinical trials in which AI-assisted management is compared with standard-of-care practice. Researchers should not only focus on demonstrating the robustness of the AI algorithm in concept studies but also on translating from code to bedside by conducting RCTs in real-life clinical settings. From our systematic review, automated polyp detection is the most widely implemented AI technology in clinical practice, which sets a good example of the pathway from algorithm development to the implementation of AI technology in real-life clinical practice. Another obstacle to the implementation of AI or ML in daily clinical practice is the regulation of these technologies [8]. To grant approval from regulatory bodies, scientific evidence is required to support the safety and effectiveness of an AI-assisted tool in clinical practice. The framework for AI health care product development highlighted that RCTs are often recommended to provide strong evidence to validate the clinical efficacy and safety of an AI-assisted tool in real-world settings [61]. More RCTs of AI-assisted tools integrated into clinical practice are required to advance the role of AI or ML in medicine. We should also test how machine intelligence and human intelligence can work together on personalized management of patients.

## Appendix

Multimedia Appendix 1 Full search strategy.

Multimedia Appendix 2 Study design and artificial intelligence (AI)–assisted tools characteristics.

## Abbreviations

AI: artificial intelligence
ML: machine learning
PRISMA: Preferred Reporting Items for Systematic Reviews and Meta-Analyses
RCT: randomized controlled trial
SPIRIT-AI: Standard Protocol Items: Recommendations for Interventional Trials-Artificial Intelligence

## Data Availability

The full search strategy is provided in Multimedia Appendix 1. Additional data are available on request.
