# Supplementary material for: Randomized Controlled Trials of Artificial Intelligence in Clinical Practice: Systematic Review
Source: J Med Internet Res. 2022 Aug 25;24(8):e37188. doi: 10.2196/37188 (PMC9459941; doi:10.2196/37188)
Supplement: Multimedia Appendix 1 [file jmir_v24i8e37188_app1.docx]

**Multimedia Appendix 1.** Full search strategy

**Medline (Ovid) search strategy**

Database: Ovid MEDLINE(R) and Epub Ahead of Print, In-Process, In-Data-Review & Other Non-Indexed Citations, Daily and Versions(R) <1946 to July 14, 2021>

Search Strategy:

--------------------------------------------------------------------------------

1 exp Artificial Intelligence/ or Deep Learning/ or exp Diagnosis, Computer-Assisted/ (188959)

2 ("artificial intelligence" or "Computational Intelligence" or "Computer Reasoning" or "Computer Vision System*" or "Knowledge Acquisition" or "Knowledge Representation" or "Machine Intelligence" or "machine learning" or "transfer learning" or "deep learning" or "Hierarchical Learning" or "computer-assisted diagnosis" or "computer assisted diagnosis").ab,ti. (67672)

3 1 or 2 (226969)

4 limit 3 to randomized controlled trial (2232)

***************************

**Embase (Ovid) search strategy**

Database: Embase <1974 to 2021 July 14>

Search Strategy:

--------------------------------------------------------------------------------

1 exp artificial intelligence/ or deep learning/ or exp computer assisted diagnosis/ (1265238)

2 ("artificial intelligence" or "Computational Intelligence" or "Computer Reasoning" or "Computer Vision System*" or "Knowledge Acquisition" or "Knowledge Representation" or "Machine Intelligence" or "machine learning" or "transfer learning" or "deep learning" or "Hierarchical Learning" or "computer-assisted diagnosis" or "computer assisted diagnosis").ab,ti. (81390)

3 1 or 2 (1314858)

4 limit 3 to randomized controlled trial (13664)

5 limit 4 to exclude medline journals (1406)

***************************

**PubMed search strategy**

| #1 | "artificial intelligence"[MeSH Terms] OR "deep learning"[MeSH Terms:noexp] OR "diagnosis, computer assisted"[MeSH Terms] | 189,079 |
| --- | --- | --- |
| #2 | "artificial intelligence"[Title/Abstract] OR "Computational Intelligence"[Title/Abstract] OR "Computer Reasoning"[Title/Abstract] OR "Computer Vision System*"[Title/Abstract] OR "Knowledge Acquisition"[Title/Abstract] OR "Knowledge Representation"[Title/Abstract] OR "Machine Intelligence"[Title/Abstract] OR "machine learning"[Title/Abstract] OR "transfer learning"[Title/Abstract] OR "deep learning"[Title/Abstract] OR "Hierarchical Learning"[Title/Abstract] OR "computer-assisted diagnosis"[Title/Abstract] OR "computer assisted diagnosis"[Title/Abstract] | 74,539 |
| #3 | #1 OR #2 | 231,978 |
| #4 | #1 OR #2 (Filters: Randomized Controlled Trial) | 2,264 |

***************************

**Cochrane Library search strategy**

Search Name:

Date Run: 14/07/2021 09:47:48

Comment:

ID Search Hits

#1 MeSH descriptor: [Artificial Intelligence] explode all trees 1128

#2 MeSH descriptor: [Deep Learning] this term only 21

#3 MeSH descriptor: [Diagnosis, Computer-Assisted] explode all trees 1867

#4 #1 OR #2 OR #3 2851

#5 ("artificial intelligence" or "Computational Intelligence" or "Computer Reasoning" or "Computer Vision System*" or "Knowledge Acquisition" or "Knowledge Representation" or "Machine Intelligence" or "machine learning" or "transfer learning" or "deep learning" or "Hierarchical Learning" or "computer-assisted diagnosis" or "computer assisted diagnosis"):ti,ab,kw (Word variations have been searched) 2723

#6 #4 OR #5 5248 (CENTRAL only: 5229)

***************************

**CINAHL search strategy**

| S1 | (MH "Diagnosis, Computer Assisted+") OR (MH "Deep Learning") OR (MH "Artificial Intelligence+") | 37,753 |
| --- | --- | --- |
| S2 | TI ( "artificial intelligence" or "Computational Intelligence" or "Computer Reasoning" or "Computer Vision System*" or "Knowledge Acquisition" or "Knowledge Representation" or "Machine Intelligence" or "machine learning" or "transfer learning" or "deep learning" or "Hierarchical Learning" or "computer-assisted diagnosis" or "computer assisted diagnosis" ) OR AB ( "artificial intelligence" or "Computational Intelligence" or "Computer Reasoning" or "Computer Vision System*" or "Knowledge Acquisition" or "Knowledge Representation" or "Machine Intelligence" or "machine learning" or "transfer learning" or "deep learning" or "Hierarchical Learning" or "computer-assisted diagnosis" or "computer assisted diagnosis" ) | 13,617 |
| S3 | S1 OR S2 | 46,188 |
| S4 | S1 OR S2 (Filters: Randomized Controlled Trial) | 708 |

***************************
