# Supplementary material for: Randomized Controlled Trials of Artificial Intelligence in Clinical Practice: Systematic Review
Source: J Med Internet Res. 2022 Aug 25;24(8):e37188. doi: 10.2196/37188 (PMC9459941; doi:10.2196/37188)
Supplement: Multimedia Appendix 2 [file jmir_v24i8e37188_app2.docx]

**Multimedia Appendix 2.** Study design and artificial intelligence (AI)–assisted tools characteristics.

| Author (publication year) | Racial information | Sample size | Setting | Design | Crossover | AI-assisted tool name | AI subtype | Data type | Dynamic data | Training data | Validation data | AI developer | AI intervention | Control |
| --- | --- | --- | --- | --- | --- | --- | --- | --- | --- | --- | --- | --- | --- | --- |
| El Solh et al [17], 2009 | Not mentioned | 115 | Single center | Single blinded | No | Not mentioned | General regression neural network | Clinical data based | Yes | Not mentioned | Not mentioned | Academic institution | ANN^a^-guided CPAP^b^ titration | Conventional CPAP titration |
| Shimabukuro et al [18], 2017 | White, African American, Asian American, Hispanic, or other | 142 | Multicenter (n=17) | Open labeled | No | InSight | Machine learning | Clinical data based | Yes | ICU^c^ stay data of 1394 patients from a single center | ICU stay data of 1394 patients from a single center | Industry | Monitored by the machine learning algorithm, in addition to the existing severe sepsis detector | Normal standard of care with monitoring by the existing EHR^d^-based severe sepsis detector |
| Labovitz et al [6], 2017 | Not mentioned | 28 | Single center | Open labeled | No | AiCure | Not mentioned | Biosignal based | No | Not mentioned | Not mentioned | Industry | Daily monitoring by AiCure | No daily monitoring by AiCure |
| Gracey et al [19], 2018 | Not mentioned | 24,527 | Not known | Double blinded | No | Not mentioned | Not mentioned | Clinical data based | No | Not mentioned | Not mentioned | Unknown | AI group was targeted to receive interventions based on AI recommendations | No intervention for control group and targeted to receive interventions based on traditional targeting methods for traditional group |
| Liu et al [20], 2018 | Not mentioned | 430 | Single center | Not known | No | Clinical Decision Support System | Machine learning | Biosignal based | No | Not mentioned | Not mentioned | Unknown | Physician assisted by a clinical decision support system | Physician without clinical decision support system |
| Vennalaganti et al [21], 2018 | Not mentioned | 160 | Multicenter (n=16) | Not known | Yes | Not mentioned | Neural network–based, high-speed computer scan | Biosignal based | No | Not mentioned | Not mentioned | Industry | WATS^e^ followed by biopsy sampling | Biopsy sampling followed by WATS |
| Biester et al [22], 2019 | Not mentioned | 34 | Multicenter (n=3) | Open labeled | Yes | DreaMed GlucoSitter | Fuzzy logic algorithms | Clinical data based | No | Not mentioned | Not mentioned | Industry | 60 hours of MD-Logic closed-loop therapy first | 60 hours of sensor-augmented pump therapy first |
| Pouska et al [23], 2019 | Not mentioned | 40 | Single center | Not known | No | Not mentioned | Machine learning | Biosignal based | Yes | Not mentioned | Not mentioned | Unknown | Usual care with hypotension probability indicator | Usual care |
| Lin et al [24], 2019 | Not mentioned | 350 | Multicenter (n=5) | Open labeled | No | Not mentioned | Deep convolutional neural network | Biosignal based | No | 410 ocular images of childhood cataracts and 476 images of normal eyes from a single center in China | 1239 cases from the same center in China, 57 cases from another 3 hospitals in China, and 53 cases from web sites | Academic institution | CC-Cruiser | Usual care by senior consultant |
| Kamdar et al [25], 2019 | Not mentioned | 112 | Single center | Not known | No | ePAL | Not mentioned | Clinical data based | No | Not mentioned | Not mentioned | Academic institution | 8 weeks usual care assisted by ePAL smartphone app | 8 weeks of usual care |
| Persell et al [26], 2020 | Not mentioned | 297 | Not known | Open labeled | No | Lark HTN^f^ Pro (beta version) | Not mentioned | Clinical data based | Yes | Not mentioned | Not mentioned | Unknown | Received an AI coaching app promoting hypertension self-management behaviors and a home monitor | Received a non-AI blood pressure tracking app and a home monitor |
| Voss et al [27], 2019 | White or European American, Black, East Asian or Asian American, South Asian or Indian American, Middle Eastern or Arab American, Hispanic or Latino or Spanish origin, or other | 71 | Single center | Open labeled | Yes | Superpower Glass | Machine learning | Biosignal based | No | Not mentioned | Not mentioned | Academic institution | Superpower glass intervention with applied behavioral analysis therapy for 6 weeks | Received only applied behavioral analysis therapy for 6 weeks |
| Wang et al [28], 2019 | Not mentioned | 1058 | Single center | Open labeled | No | EndoScreener | Deep convolutional neural network | Biosignal based | No | 5545 colonoscopy images of 1290 patients from a single center in China | Data sets A (27,113 colonoscopy images of 1138 patients), C (138 colonoscopy videos of 110 patients), and D (54 unaltered colonoscopy videos of 54 patients) are from the same center in China and data set B (612 colonoscopy images) is from a center in Spain | Industry | CADe^g^ colonoscopy | Routine colonoscopy |
| Wu et al [29], 2019 | Not mentioned | 324 | Single center | Single blinded | No | WISENE (precursor of ENDOANGEL) | Deep convolutional neural network and deep reinforcement learning | Biosignal based | No | 88,715 EGD^h^ images of >3000 patients from a single center in China | 107 EGD videos from a single center in China | Academic institution | EGD exam with the assistance of WISENSE | EGD exam without the assistance of WISENSE |
| Pavel et al [30], 2020 | Not mentioned | 258 | Multicenter (n=8) | Open labeled | No | Algorithm for Neonatal Seizure Recognition | Support vector machine | Biosignal based | No | Not mentioned | Not mentioned | Academic institution | Continuous EEG^i^ monitoring with the aid of Algorithm for Neonatal Seizure Recognition | Routine continuous EEG monitoring alone |
| Alfonsi et al [31], 2020 | Not mentioned | 46 | Single center | Not known | No | Not mentioned | Convolutional neural network | Biosignal based | No | Not mentioned | Not mentioned | Industry | Usual care with AI iSpy app | Usual care |
| Auloge et al [32], 2020 | Not mentioned | 20 | Single center | Open labeled | No | Prototype of an investigational device developed by Philips Healthcare | Not mentioned | Biosignal based | No | Not mentioned | Not mentioned | Industry | AR^j^ or AI-guidance with motion compensation | Standard fluoroscopic guidance |
| Avari et al [33], 2020 | Not mentioned | 50 | Multicenter (n=2) | Open labeled | Yes | PEPPER | Case-based reasoning | Clinical data based | No | Not mentioned | Not mentioned |  | PEPPER or control | Control or PEPPER |
| Chen et al [34], 2020 | Not mentioned | 214 | Single center | Single blinded | No | ENDOANGEL | Deep convolutional neural networks | Biosignal based | No | 88,715 EGD images of >3000 patients from a single center in China | 107 EGD videos from a single center in China | Academic institution | Sedated conventional EGD, unsedated conventional EGD, and unsedated ultrathin transoral endoscopy with assistance of ENDOANGEL | Sedated conventional EGD, unsedated conventional EGD, and unsedated ultrathin transoral endoscopy without assistance of ENDOANGEL |
| Gong et al [35], 2020 | Not mentioned | 704 | Single center | Single blinded | No | ENDOANGEL | Deep convolutional neural networks and random forest model | Biosignal based | No | 34,871 colonoscopic images from a single center in China | 4600 colonoscopy images and 325 colonoocopy videos from a single center in China | Academic institution | ENDOANGEL-assisted colonoscopy | Unassisted colonoscopy |
| Liu et al [36], 2020 | Not mentioned | 790 | Single center | Open labeled | No | EndoScreener | Deep convolutional neural network | Biosignal based | No | 5545 colonoscopy images of 1290 patients from a single center in China | Data sets A (27,113 colonoscopy images of 1138 patients), C (138 colonoscopy videos of 110 patients), and D (54 unaltered colonoscopy videos of 54 patients) are from the same center in China and data set B (612 colonoscopy images) is from a center in Spain | Industry | Routine colonoscopy with the assistance of a real-time polyp detection CADe system | Routine colonoscopy without the assistance of a real-time polyp detection CADe system |
| Nicolae et al [37], 2020 | Not mentioned | 41 | Single center | Single blinded | No | Not mentioned | Machine learning | Clinical data based | No | 100 high-quality cases | Not mentioned | Academic institution | Treatment planning using a machine learning–based PIPA^k^ system | Conventional treatment planning |
| Repici et al [38], 2020 | Not mentioned | 700 | Multicenter (n=3) | Single blinded | No | GI-Genius | Convolutional neural network | Biosignal based | No | A series of videos of 2346 histologically confirmed polyps of 735 patients from 20 centers in Europe and the United States | 338 polyps of 105 patients from 20 centers in Europe and the United States | Industry | Colonoscopy with CADe | Colonoscopy without CADe |
| Su et al [39], 2020 | Not mentioned | 623 | Single center | Single blinded | No | AQCS^l^ | Deep convolutional neural network and deep learning framework | Biosignal based | No | 15,951 endoscopic images of >4000 patients from a single center in China | 7661 endoscopic images of >4000 patients and 30 colonoscopy videos from a single center in China | Academic institution | Routine colonoscopy with AQCS | Routine colonoscopy without AQCS |
| Wang et al [40], 2020 | Not mentioned | 369 | Single center | Open labeled | Yes | EndoScreener | Deep convolutional neural network | Biosignal based | No | 5545 colonoscopy images of 1290 patients from a single center in China | Data sets A (27,113 colonoscopy images of 1138 patients), C (138 colonoscopy videos of 110 patients), and D (54 unaltered colonoscopy videos of 54 patients) are from the same center in China and data set B (612 colonoscopy images) is from a center in Spain | Industry | CAD-colonoscopy first | Routine colonoscopy first |
| Wang et al [41], 2020 | Not mentioned | 962 | Single center | Double blinded | No | EndoScreener | Deep convolutional neural network | Biosignal based | No | 5545 colonoscopy images of 1290 patients from a single center in China | Data sets A (27,113 colonoscopy images of 1138 patients), C (138 colonoscopy videos of 110 patients), and D (54 unaltered colonoscopy videos of 54 patients) are from the same center in China and data set B (612 colonoscopy images) is from a center in Spain | Industry | Colonoscopy with polyp CADe system | Colonoscopy with sham system |
| Wijnberge et al [42], 2020 | Not mentioned | 60 | Single center | Open labeled | No | Early warning system software | Logistic regression model | Biosignal based | Yes | 1334 patients’ records with 545,959 minutes of arterial waveform recording and 25,461 episodes of hypotension from a single center in the Netherlands | 204 patients’ records with 33,236 minutes of arterial waveform recording and 1923 episodes of hypotension from a single center in the Netherlands | Industry | Intraoperative early warning system | Standard care |
| Weisinger et al [43], 2021 | Not mentioned | 25 | Not known | Double blinded | No | BrainQ | Not mentioned | Biosignal based | Yes | Not mentioned | Not mentioned | Industry | Artificial intelligence–powered, frequency-tuned ELF-EMF^m^ treatment (BQ) | Sham |
| Blomberg et al [44], 2021 | Not mentioned | 654 | Single center | Double blinded | No | Not mentioned | A network of several machine learning models | Biosignal based | No | Not mentioned | Not mentioned | Academic institution | A machine learning model that analyzes calls in real time and delivers decision support | Conventional call-handling without decision support from the machine learning model |
| Browning et al [45], 2021 | White or ethnic minority | 778 | Multicenter (n=76) | Open labeled | No | Not mentioned | Linear support vector machine | Clinical data based | No | Not mentioned | Not mentioned |  | Antidepressant treatment guided by a predictive algorithm | Treatment as usual |
| Jayakumar et al [46], 2021 | White, Asian, Black or African American, or Hispanic or Latino | 129 | Single center | Open labeled | No | Joint Insights | Not mentioned | Clinical data based | No | Not mentioned | Not mentioned | Industry | Receives decision aid including patient education, preference assessment, and personalized outcome estimations | Receives educational material only alongside usual care |
| Kamba et al [47], 2021 | Not mentioned | 358 | Multicenter (n=4) | Not known | Yes | Not mentioned | Deep convolutional neural network | Biosignal based | No | 65,421 colonoscopic images of 4147 patients including 26,729 lesions from a single center in Japan | 4158 colonoscopic images from a single center in Japan | Academic institution | CADe-assisted colonoscopy first and then standard colonoscopy | Standard colonoscopy first and then CADe-assisted colonoscopy |
| Luo et al [48], 2021 | Not mentioned | 300 | Single center | Not known | Yes | Eagle Eye | Convolutional neural network | Biosignal based | No | 112,199 colonoscopy images from a single center in China | Not mentioned | Industry | AI-assisted colonoscopy first, followed by traditional colonoscopy, no wash out period, and back to back | Traditional colonoscopy first, followed by AI-assisted colonoscopy, no wash out period, and back to back |
| Rafferty et al [49], 2021 | Not mentioned | 25 | Single center | Open labeled | No | Heali | Not mentioned | Clinical data based | No | Not mentioned | Not mentioned | Industry | Received access to the mobile app and educational materials | Received educational materials |
| Repici et al [50], 2021 | Not mentioned | 660 | Multicenter (n=5) | Not known | No | GI-Genius | Convolutional neural network | Biosignal based | No | A series of videos of 2346 histologically confirmed polyps of 735 patients from 20 centers in Europe and the United States | 338 polyps of 105 patients from 20 centers in Europe and the United States | Industry | CADe in real-time colonoscopy | Traditional colonoscopy |
| Strömblad et al [51], 2021 | Asian, Black, White, or other | 683 | Single center | Not known | No | Not mentioned | Not mentioned | Clinical data based | No | Not mentioned | Not mentioned | Academic institution | Machine learning–assisted model | Scheduling-flow system |
| Wu et al [52], 2021 | Not mentioned | 1002 | Multicenter (n=5) | Not known | No | ENDOANGEL | Deep convolutional neural networks and deep reinforcement learning | Biosignal based | No | 88,715 EGD images of >3000 patients from a single center in China | 107 EGD videos from a single center in China | Academic institution | EGD with ENDOANGEL assistance | EGD without ENDOANGEL assistance |
| Yao et al [53], 2021 | White, Black or African American, Asian, or other | 22,641 | Multicenter (n=45) | Single blinded | No | Not mentioned | Convolutional neural network | Biosignal based | No | 35,970 patients with paired ECGs^n^ and echocardiograms | 8989 patients with paired ECGs and echocardiograms | Academic institution | Usual care with access to AI results | Usual care with no access to AI results |
| Brown et al [54], 2021 | Asian, African American, White (non-Hispanic), or other | 232 | Multicenter (n=4) | Single blinded | Yes | EndoScreener | Deep convolutional neural network | Biosignal based | No | 5545 colonoscopy images of 1290 patients from a single center in China | Data sets A (27,113 colonoscopy images of 1138 patients), C (138 colonoscopy videos of 110 patients), and D (54 unaltered colonoscopy videos of 54 patients) are from the same center in China and data set B (612 colonoscopy images) is from a center in Spain | — | Colonoscopy with the assistance of a real-time polyp detection CADe system first | High-definition white light colonoscopy without the assistance of a real-time polyp detection CADe system first |

^a^ANN: artificial neural network.

^b^CPAP: continuous positive airway pressure.

^c^ICU: intensive care unit.

^d^EHR: electronic health record.

^e^WATS: wide-area transepithelial sampling.

^f^HTN: hypertension.

^g^CADe: computer-aided detection.

^h^EGD: esophagogastroduodenoscopy

^i^EEG: electroencephalogram.

^j^AR: augmented reality.

^k^PIPA: prostate implant planning algorithm.

^l^AQCS: automatic quality control system.

^m^ELF-EMF: extremely low frequency and low intensity electromagnetic fields

^n^ECG: electrocardiogram.
